# Cell Size at S Phase Initiation: An Emergent Property of the G_1_/S Network

**DOI:** 10.1371/journal.pcbi.0030064

**Published:** 2007-04-13

**Authors:** Matteo Barberis, Edda Klipp, Marco Vanoni, Lilia Alberghina

**Affiliations:** 1 Department of Biotechnology and Biosciences, University of Milano-Bicocca, Milan, Italy; 2 Max-Planck Institute for Molecular Genetics, Computational Systems Biology, Berlin, Germany; University of California San Diego, United States of America

## Abstract

The eukaryotic cell cycle is the repeated sequence of events that enable the division of a cell into two daughter cells. It is divided into four phases: G_1_, S, G_2_, and M. Passage through the cell cycle is strictly regulated by a molecular interaction network, which involves the periodic synthesis and destruction of cyclins that bind and activate cyclin-dependent kinases that are present in nonlimiting amounts. Cyclin-dependent kinase inhibitors contribute to cell cycle control. Budding yeast is an established model organism for cell cycle studies, and several mathematical models have been proposed for its cell cycle. An area of major relevance in cell cycle control is the G_1_ to S transition. In any given growth condition, it is characterized by the requirement of a specific, critical cell size, P_S_, to enter S phase. The molecular basis of this control is still under discussion. The authors report a mathematical model of the G_1_ to S network that newly takes into account nucleo/cytoplasmic localization, the role of the cyclin-dependent kinase Sic1 in facilitating nuclear import of its cognate Cdk1-Clb5, Whi5 control, and carbon source regulation of Sic1 and Sic1-containing complexes. The model was implemented by a set of ordinary differential equations that describe the temporal change of the concentration of the involved proteins and protein complexes. The model was tested by simulation in several genetic and nutritional setups and was found to be neatly consistent with experimental data. To estimate P_S_, the authors developed a hybrid model including a probabilistic component for firing of DNA replication origins. Sensitivity analysis of P_S_ provides a novel relevant conclusion: P_S_ is an emergent property of the G_1_ to S network that strongly depends on growth rate.

## Author Summary

A major property of living cells is their ability to maintain mass homeostasis throughout cell divisions. It has been proposed that in order to achieve such homeostasis, some critical event(s) in the cell cycle will take place only when the cell has grown beyond a critical cell size. In the budding yeast *Saccharomyces cerevisiae,* a widely used model for the study of the eukaryotic cell cycle, a large body of evidence indicates that cells have to reach a critical size before they start to replicate their DNA and to form bud, which will give rise to the daughter cell. This critical cell size is modulated by growth rate, hence by nutritional conditions and the multiplicity of genetic material (i.e., ploidy). The authors present a mathematical model of the regulatory molecular network acting at the G_1_ to S transition. The major novel features of this model compared with previous models of this process are (1) the accounting for cell growth (i.e., the increase in cell volume); (2) the explicit consideration of the fact that cells have a nucleus and a cytoplasm, and that key cell cycle regulatory molecules must move between these different compartments and can only react or regulate each other if they are in the same compartment; and (3) the requirement of sequential overcoming of two molecular thresholds given by a cyclin-dependent kinase/cyclin and a cyclin-dependent kinase inhibitor. The model was tested by simulating the processes during G_1_ to S transition for different growth conditions or for different mutants and by comparing the results with experimental data. A parameter sensitivity analysis (i.e., testing the model predictions when parameters are varied), newly indicates that the critical cell size is an emergent property of the G_1_ to S network. The model leads to a unified interpretation of seemingly disparate experimental observations and makes predictions to be experimentally verified.

## Introduction

During the life cycle of eukaryotic cells, DNA replication is restricted to a specific time window, called the S phase. Several control mechanisms ensure that each DNA sequence is replicated once, and only once, in the period from one cell division to the next. Following S phase, replicated chromosomes separate during mitosis (M phase) and segregate in two nuclei that eventually will be endowed to each newborn daughter cell at cell division. Two gap phases, called G_1_ and G_2_, separate cell birth from S phase and S phase from M phase, respectively.

**Figure 1:**
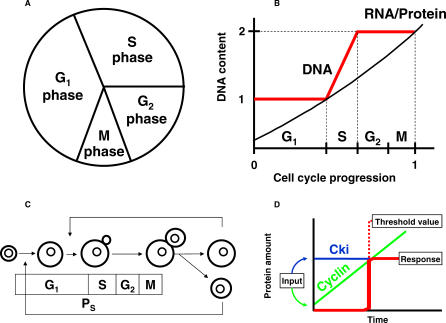
Main Events That Occur during the Yeast Cell Cycle (A) General representation of the cell cycle showing the discontinuous events that have to take place only once per cell cycle, namely the S phase and the M phase, spaced with G_1_ and G_2_ phases that allow increase of the cell size before DNA replication and cell division, respectively. (B) During the dynamics of the cell cycle, RNA and proteins increase exponentially, while the DNA content show a typical doubling amount until the cell divides to generate a newborn daughter. From G_1_ to M phases, the cell increases continuously in mass. (C) Typical representation of the cell cycle that points out the coordination of the increase in cell mass with DNA replication and cell division in order to maintain size homeostasis. DNA replication and cell division start only when cells have reached a critical cell size (P_S_ and P_M_, respectively). (D) General representation of the molecular threshold. It involves two molecules, an activator and an inhibitor. When the activator increases with growth, the threshold is overcome when enough molecules of the activator are made to exceed the inhibitor.

Typical pie chart representation of the cell cycle (Figure 1A) stresses the discontinuous events that have to take place only once per cell cycle (i.e., S and M phases), but fails to show that proliferating somatic cells are continuously increasing in their mass throughout the cell cycle (Figure 1B). As pointed out as early as 1971 by Mitchinson [1], the “continuous events of the growth cycle” (i.e., increase in cell mass) and the “discontinuous events of the DNA division cycle” (i.e., DNA replication, mitosis, and cell division) need to be tightly coordinated in order to maintain cell size homeostasis. It has been proposed that coordination of mass accumulation with cell cycle progression relies on a sizer mechanism, so that DNA replication and/or cell division start only when cells have reached a critical cell size (see [2] for a review). In this way, tiny newborn cells will have to grow more than mother cells before being able to overcome the cell size checkpoint. Conversely, a larger cell will overcome the cell size checkpoint earlier than the “normal, average” cell. As a result, both small and large cells will stabilize cell size to the “normal, average” value (Figure 1C).

Although evidence for the occurrence of a sizer mechanism(s) has been collected in different eukaryotes from unicellular microorganisms to flies to mammalian cells [3,4], cell size control has been best studied and come to be generally accepted in unicellular fungi, such as the distantly related fission and budding yeasts. In the fission yeast *Schizosaccharomyces pombe,* a size control is operative over mitosis in wild-type cells, while a cryptic control over S phase is revealed in *wee* mutants [5] or under conditions of nutritional limitation [6]. In the budding yeast *S. cerevisiae,* the major size control is operative over DNA replication and bud emergence (G_1_/S transition, often referred to as “Start” in this organism). We refer to the critical cell size required for the G_1_/S transition in budding yeast as P_S_, which represents the protein content per cell at the onset of DNA replication. Ploidy, nutrients, and growth rate modulate P_S_ that is smaller in slow-growing cells than in fast-growing cells [2,7].

Despite extensive efforts, the molecular basis of the cell sizer mechanism controlling the G_1_/S transition in budding yeast has long remained elusive. While gene dosage data strongly argued for a role for Cln3 in the mechanism [8,9], physiological data gave apparently contradictory results [10–12]. We propose that a growth-dependent threshold lies at the core of the cell sizer mechanism. In its essence, a threshold entails the interplay of two molecules, one acting as an activator and a second acting as an inhibitor [13,14]. When the number of molecules of activator is low and that of inhibitor is high, the activator is below the threshold; if the activator increases with growth, the threshold is overcome when enough molecules of activator are made to exceed the inhibitor. This mechanism is schematically reported in Figure 1D under the idealized assumption that the equilibrium is totally shifted toward the formation of the complex. We propose that the activator and inhibitor molecules are, respectively, Cln3 and Far1 [14,15].

Understanding of the molecular basis of the critical cell size–controlling onset of S phase (P_S_) would be of great relevance for cell biology. To this end, we present here a mathematical model of the G_1_ to S transition that integrates the different regulatory links proposed so far. The model takes into consideration data from the literature and various models (not necessarily mathematical models) that were proposed for the G_1_ to S transition [2,3,16], together with our own recent experimental results [15,17]. The model was implemented by a set of ordinary differential equations (ODEs) [18], an approach successfully used to describe the cell cycle control in budding yeast [19,20] and the cellular response of yeast to hyperosmotic shock [21]. These equations describe the temporal change of the concentrations of the involved proteins and complexes. Our model explicitly considers the localization of components in different cellular compartments (cytoplasm or nucleus) and cell growth during the G_1_ phase. We investigate the dynamics of the G_1_/S transition in various growth conditions, in several mutants, and in response to different signaling pathways. Sensitivity analysis in the form of time-dependent response coefficients [22] is used to estimate the influence of parameter values on the dynamics of key components and to investigate the relevance of the nucleo/cytoplasmic localization of Sic1 on the regulation of the G_1_/S transition, which has been neglected by earlier cell cycle models. To arrive at experimentally testable predictions, we developed two extended versions of the model. To compare the model output with data on budding, stochasticity in parameter values was taken into account. To estimate the P_S_ value, we constructed a hybrid model regarding the firing of DNA replication origins where the probabilistic model uses as input the output of the deterministic model, here the nuclear concentration of Cdk1-Clb5,6. Our results newly indicate that P_S_ is an emergent property of the network that depends on growth rate, thereby rationalizing the observed increase of P_S_ at fast growth rates.

## Results

The goal of the present work is to simulate the dynamics of the G_1_ to S transition of the budding yeast cell cycle. Starting from the literature data, we have built a mathematical model describing the main molecular events involved and tested it in two different experimental conditions (growth of the cells on glucose and on ethanol media). We have verified the reliability of the model by simulation of the effect of altering gene dosage of the major actors involved, by analyzing the behavior of a large number of mutants, and by simulating the response to various signaling pathways (the pheromone pathway and the stress-response Hog1-dependent pathway). We have further considered the heterogeneity of the cell population, and simulated its dynamics. Finally, we have estimated from the model the critical cell size that cells require to enter S phase both in glucose and in ethanol growth conditions, and in a number of relevant mutants growing in glucose.

### Data Mining and Wiring for Molecular Events of the G_1_ to S Transition

In an effort to cope with the recently released recommendations for annotation of biochemical models [23], we first list experimental data used for designing the network structure. In Materials and Methods we describe the modeling principles [18,21] and give details on parameter estimation. The full set of equations is given in Table 1, while values of parameters are summarized in Tables 2–5.

#### Regulation of Cdk1-Cln3.

Cln3 is the most upstream cyclin in cell cycle progression [24]. Its concentration is low compared with other cyclins and remains roughly constant during G_1_ [24–26]. Growth conditions modulate the level of Cln3 that is higher in fast-growing cells and lower in slow-growing cells [11]. The level of Cdk1 is very large and roughly constant during the cell cycle [27], and its activation by Cak1 does not appear to be limiting, so Cak1 has been omitted in our model. In growing cells, Cln3, which is largely nuclear [28], forms the binary complex Cdk1-Cln3.

The Cdk inhibitor (Cki) Far1, whose role in mitotic cell cycle has been ascertained recently [15,29], is largely enriched in newborn cells by a burst of synthetic activity at the end of the previous cycle, and its amount per cell is roughly constant during the G_1_ phase [30]. It is assumed that Far1 binds to the Cdk1-Cln3 complex. Given the presence of a substantial amount of Far1 in the cell, in newborn cells the inhibitory interaction between Far1 and Cln3 traps Cdk1 in the inactive ternary complex Cdk1-Cln3-Far1. Growth-dependent accumulation of Cln3 allows it to overcome the threshold set by Far1, freeing the active Cdk1-Cln3 complex. Far1 is phosphorylated (at serine 87) by Cdk1-Cln complexes [31], and hence is primed for degradation, yielding a substantial amount of free active Cdk1-Cln3.

#### Regulation of transcription factor activity.

Active Cdk1-Cln3 activates the transcription factors SBF and MBF, whose basal activity is kept low by Whi5 [32,33]. Cdk1-Cln3 phosphorylates Whi5, promoting its dissociation from SBF and, possibly, its nuclear export. When freed from Whi5, SBF activates *CLN1,2* transcription, thereby promoting synthesis of the encoded proteins. MBF similarly promotes synthesis of Clb5 and Clb6 [34]. The activation of SBF and MBF by Cdk1-Cln3 commits a cell to DNA replication and budding (i.e., to a new cell cycle). When no Cln3 is present, other cyclins, such as Bck2, may substitute for it, although quite inefficiently; therefore, the entrance into S phase takes place at a larger cell size and after a longer G_1_ phase than in wild-type cells [35]. This salvage pathway is accounted for by inclusion of a low, basal (i.e., Cln3-independent) rate of SBF/MBF activation.

#### Regulation of Cdk1-Cln1,2 and Cdk1-Clb5,6.

In the cytoplasm, Cln1 and Cln2 bind to Cdk1 and, thereby, promote the biochemical steps that result in budding [36]. The process of budding is described with a simple probabilistic model (see below). Cytoplasmic Clb5 and Clb6 bind to Cdk1, and the newly formed complex Cdk1-Clb5,6 is inhibited by cytoplasmic Sic1. Like Far1, Sic1 is largely accumulated in the newborn cell being synthesized at anaphase/telophase [37]. Therefore, active Cdk1-Clb5,6 complexes are released only after the overcoming of the second threshold set by Sic1. Sic1 degradation is primed by multisite phosphorylation by Cdk1-Cln1,2 [38]. Active Cdk1-Clb5,6 complexes are responsible for initiating DNA synthesis [39].

#### Sic1 localization.

Sic1 is produced in the cytoplasm, where it binds to Cdk1-Clb5,6, favoring its nuclear import [17]. In G_1_ cells growing in glucose-supplemented media, Sic1 is mostly nuclear, while in ethanol-grown cells a large amount of Sic1 remains cytoplasmic [17]. Sic1 parameters have been set to account for this differential localization (Tables 2–5) applying the known binding affinity of Sic1 to the cyclin-dependent kinase Cdk–cyclin complex “in vitro” [40,41] to simulation of glucose-grown cells, and estimating that for ethanol-grown cells as reported in Materials and Methods.

#### Onset of DNA replication.

DNA replication starts at multiple replication origins (about 300–500/genome) [42,43] set in regular fashion along the chromosomes. During late M and early G_1_ phases, a multiprotein pre-replicative complex assembles at each replication origin. The activation of a pre-replicative complex during S phase is quite complex [39], but a relevant step is the Cdk1-Clb5,6 phosphorylation of different substrates that induces firing of the DNA replication origins [39]. We describe these steps with a simple probabilistic model that links the availability of Cdk1-Clb5,6 nuclear concentration to origin firing.

**Table 1:**
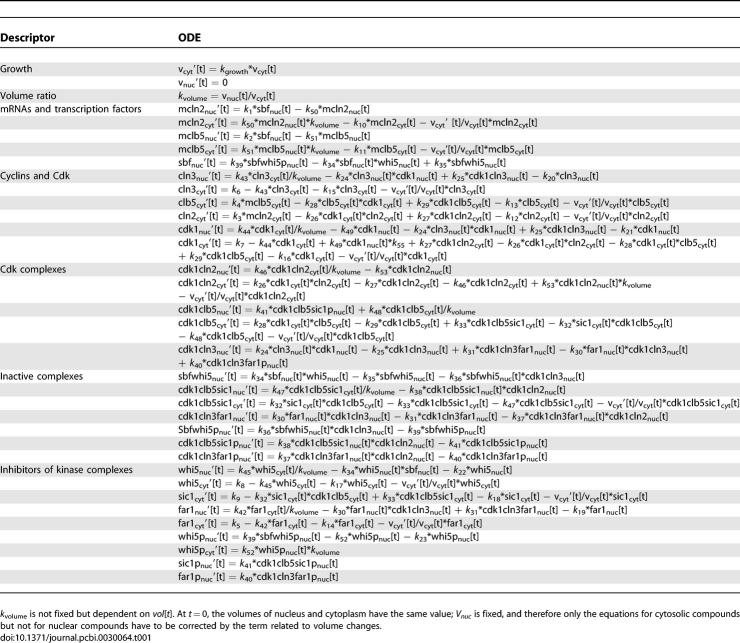
Set of ODEs Describing the Systems Dynamics

### Structure of the Network Controlling the G_1_ to S Transition in Budding Yeast

**Figure 2:**
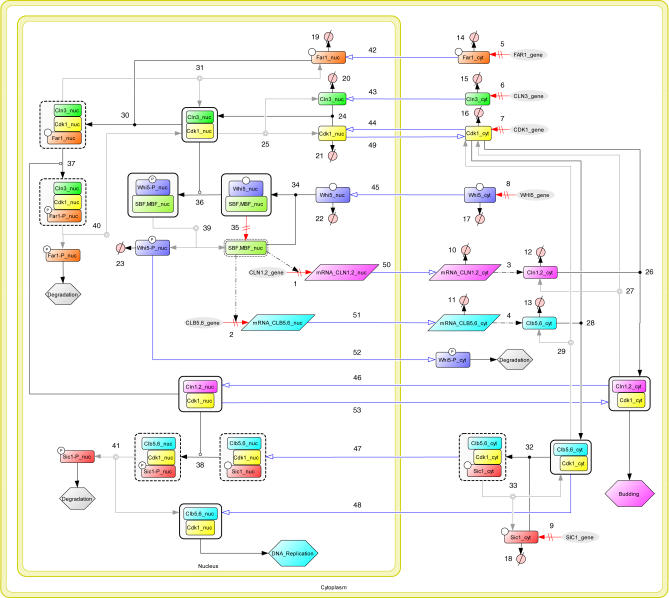
Processes Regulating the G_1_/S Transition in Yeast Cell Cycle The model comprises transcription of genes coding for cyclins (reactions 1–2), mRNA translation for cyclins, Cdk1, and Ckis (3–9), degradation of mRNA (10–11) and proteins (12–23), reversible or irreversible formation of binary (24–29) and ternary (30–34) protein complexes, Cln3-independent formation of SBF/MBF (35), phosphorylation of protein complexes (36–38), and dissociation of phosphorylated protein complexes (39–41) followed by degradation of the phosphorylated protein. Transport of proteins and protein complexes occurs from cytoplasm to nucleus (42–48) and vice versa (49–53).

To study the G_1_ to S transition network, we assembled all the essential elements into a concise mathematical model that captures the logic of the underlying processes. The main goal is to represent as much complexity as possible through a small number of quantities that have direct experimental interpretation. We drew the model with CellDesigner [44], a structured diagram editor for drawing gene-regulatory and biochemical networks that are stored using the Systems Biology Markup Language (SBML), a standard for representing models of biochemical and gene-regulatory networks. The essential elements we considered are (Figure 2): (1) production and degradation of mRNAs and proteins; (2) formation of dimeric and trimeric protein complexes; (3) nucleo/cytoplasmic localization of the compounds, transport processes being described like reactions, (e.g., converting Cdk1_cyt_ into Cdk1_nuc_); (4) cell growth in terms of volume increase with proportional rate constants for growth and protein production; and hence (5) concentration changes in the nuclear and cytoplasmic compartments. We use these elements to develop a mathematical model based on ODEs that describes the dynamics of both how different molecular species transform into each other, and how they traffic between the cytoplasmic and nuclear compartments (Table 1).

**Table 2:**
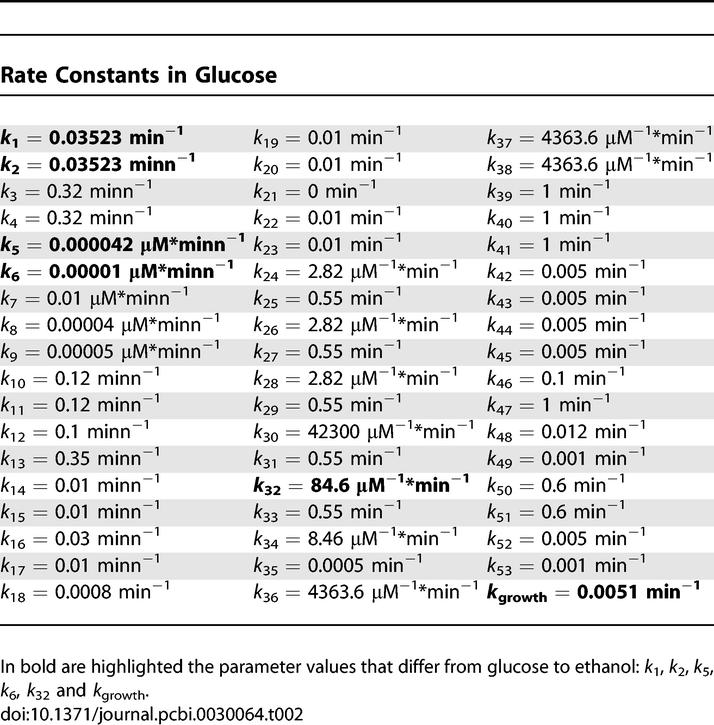
Rate Constants in Glucose Medium

In its essence, the time course of the network can be summarized as follows (see paragraphs above for references). The first threshold is based on the growth-dependent cyclin Cln3, and on the Cki Far1. Cln3 concentration remains approximately constant during G_1_, so that its total amount in the cell increases proportional to cell mass. Given that Far1 is mostly endowed to the newborn cell at the end of the previous cycle, the cell sizer threshold will be activated when Cln3 overcomes Far1. The ensuing Cdk1-Cln3–catalyzed release of the inhibitory effect exerted by Whi5—a protein with a role similar to that of pRb in mammalian cell cycle—on SBF/MBF will promotes synthesis of Cln1,2 and Clb5,6. Far1 is degraded after priming by Cdk1-Cln1,2, thus introducing a reinforcing loop in the network that guarantees irreversibility to the transition. The second threshold Cdk1-Clb5,6/Sic1 is overcome when Sic1 gets degraded following Cdk1-Cln1,2–primed phosphorylation.

**Table 3:**
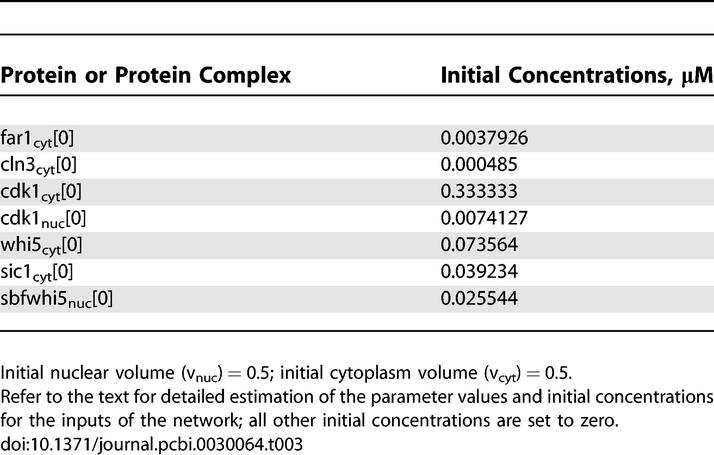
Initial Concentrations in Glucose Medium

**Table 4:**
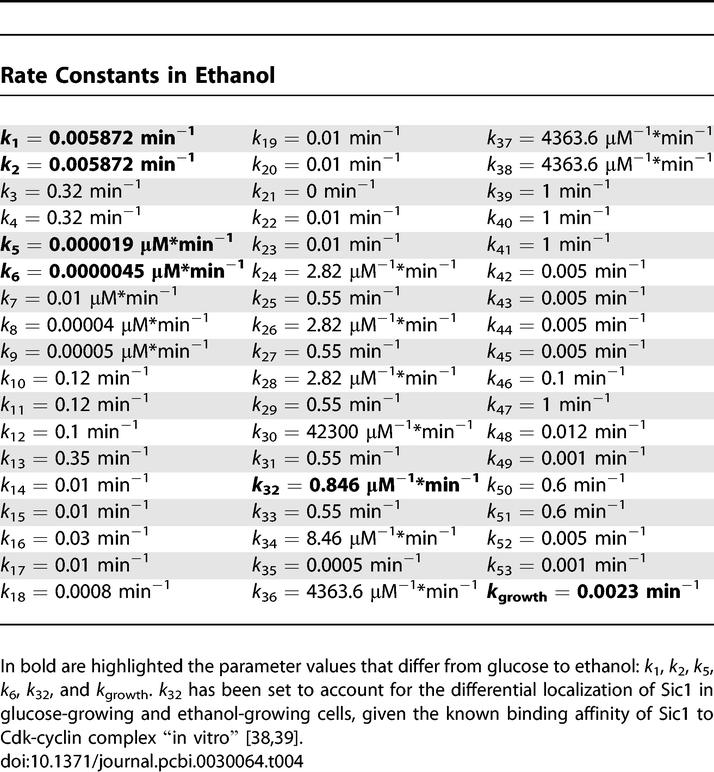
Rate Constants in Ethanol Medium

### Outline of the Computational Analysis

The model was constrained to fit the observed experimental behavior of the G_1_ to S transition of small, elutriated G_1_ cells growing on glucose [15,17]. First, we constrained features that play important regulatory roles in this phase of the cell cycle, such as the Cln3/Far1 threshold that controls the release of the SBF and MBF transcription factors from the Whi5 transcriptional repressor, and the complex formation of Sic1 with Cdk1-Clb5,6. To simulate cell growth at a low growth rate (in ethanol medium), we used both parameters' values derived from experimental data (such as the initial levels of Cln3 and Far1 and the growth rate), and a parameter (the binding constant of Sic1 for the Cdk1-Clb5,6 complex) inferred from the experimental dynamics of Clb5 and Sic1 as indicated in the following. A sensitivity analysis was performed for the fine-tuning of the parameters used to simulate the biochemical network. Then, we tested the mathematical model with the dynamics of (1) a large number of mutants, implemented by overexpression or deletion of key regulatory genes; (2) time course of budding obtained both in glucose and in ethanol media; and (3) estimation of the critical cell size P_S_ for wild-type cells grown in both media. Finally, a sensitivity analysis indicates that P_S_ is an emergent property of the G_1_ to S network.

**Table 5:**
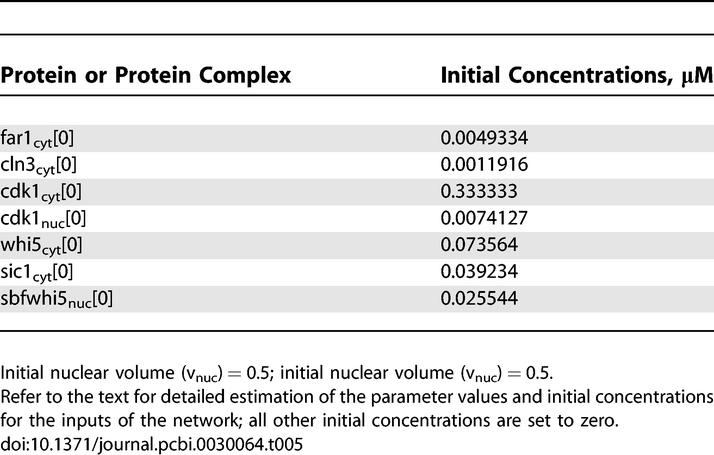
Initial Concentrations in Ethanol Medium

### Dynamics of Key Players During the G_1_ to S Transition

**Figure 3:**
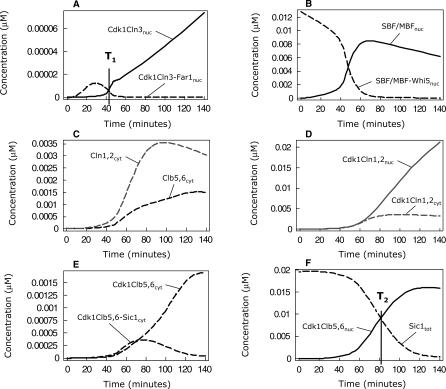
Simulated Time Courses for Protein and Protein Complex Concentrations *T_1_* and *T_2_* represent the threshold-overcome times. In early G_1_, cytoplasmic Far1, Cln3, and Cdk1 are imported into the nucleus. (A) Nuclear Cdk1-Cln3-Far1 complex reaches its maximal value after 30 min and becomes degraded upon overcoming the Cln3/Far1 threshold (*T*_*1*_). Then, Cdk1-Cln3 accrues in the nucleus. (B) At about 50 min the accumulation of active SBF/MBF reaches its half-maximal value. (C) Cln and Clb cyclins are produced in substantial amounts. (D) Cdk1-Cln1,2 is accumulated both in cytoplasm and nucleus. (E–F) Cdk1-Clb5,6 accumulates preferentially in the nucleus. (F) The half-maximal value of nuclear Cdk1-Clb5,6 is reached at around 80 min, thereby setting the value of the second threshold (*T_2_*).

Figure 3 displays the simulated time courses of the concentration of several cell cycle players in small newborn cells growing in glucose using the following input parameters: growth rate characteristic of glucose; cell size; and Cln3 and Far1 levels as detected in small, elutriated cells grown in the same medium [15]. Since we are simulating the G_1_/S transition and not a full cell cycle with all relevant proteins, time courses of some variables become meaningless after overcoming the second threshold.

At the beginning, the Far1, Cln3, and Cdk1 present in the cytoplasm are imported in the nucleus. The Cdk1-Cln3-Far1 complex reaches its maximal value in the nucleus after 30 min, and then it starts to be degraded upon the overcoming of the Cln3/Far1 threshold. Such degradation is mostly dependent on the execution of the first threshold, being strongly delayed in simulated *cln3*Δ cells (unpublished data). Cdk1-Cln3 starts to build up in the nucleus, the major factor driving accumulation of Cdk1-Cln3 being Cdk1-Cln–primed Far1 degradation (Figure 3A; *T_1_*), since little if any Cdk1-Cln3 complex forms in *cln1,2*Δ cells (unpublished data). At about 50 min, the accumulation of active SBF/MBF reaches its half-maximal value (Figure 3B). Cln and Clb cyclins are produced (Figure 3C), and Cdk1-Cln1,2 is accumulated both in cytoplasm and in nucleus (Figure 3D), while Cdk1-Clb5,6 accumulates preferentially in the nucleus (Figure 3E and 3F). The half-maximal value of Cdk1-Clb5,6 in the nucleus is reached at around 80 min (Figure 3F; *T_2_*). Thus, *T_1_* and *T_2_* represent the times when the first and second thresholds, respectively, are overcome. The coherence of this timing with experimental dynamics of cell cycle is discussed below.

### Testing the Performance of the Model

#### Simulation of the G_1_ to S transition in ethanol-grown cells.

**Figure 4:**
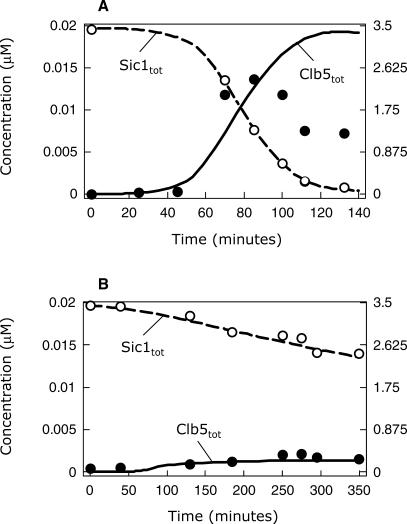
Comparison of Experimental Data and Simulation Results for Total Sic1 and Clb5 Experimental protein levels, indicated as white (Sic1) and black (Clb5) dots, were determined for elutriated wild-type cells grown in glucose medium (A) or ethanol medium (B). For further explanation, see text.

The simulation analyses performed so far have considered only cells grown on glucose medium. To assess the effect of changing growth conditions from glucose to ethanol media on the simulated G_1_ to S transition, we needed to change input parameters such as the growth rate and the initial levels of Cln3 and Far1. Besides, since it is known that in G_1_ cells grown in ethanol, a large portion of Sic1 is in the cytoplasm, whereas in G_1_ cells grown in glucose Sic1 is completely nuclear [17], we needed to also model this differential localization. To do so, we considered the fitting between experimental and simulated time course of total Sic1 and Clb5 (Figure 4). The agreement of experimental and simulated dynamics of Sic1 and Clb5 was very good for cells growing in glucose (Figure 4A). For Clb5, the match is satisfactory until about 90 min. The simulation does not cover the subsequent decrease in experimental data (Figure 4A; last three black dots), since the components that ensure the downregulation of Clb5 after S phase (the accumulation of Cdk1-Clb3,4) are not considered in the present model.

Experimental and simulated time courses of Sic1 and Clb5 levels of elutriated cells grown on ethanol are reported in Figure 4B. To obtain a good fitting of experimental and simulated dynamics in ethanol, it was not enough to change the growth rate and the Cln3 and Far1 levels—according to reported results [15]—but it required, in addition, a large change (i.e., the reduction by two orders of magnitude) of the binding constant of Sic1 to the Cdk1-Clb5,6 complex (see Tables 2–5 for comparison). The assumption that the binding constant between two proteins could be affected by the growth conditions of the cells is consistent with many data reported in the literature. In fact, it is known that phosphorylation of a protein can affect binding to another protein [45], and that changes of the physiological state of a cell often generate signals that change the phosphorylation state of target proteins. Moreover, Barberis et al. reported that a Sic1-derived peptide has a substantially increased affinity towards a heterologous Cdk-cyclin complex after phosphorylation by CK2 kinase, compared with that of the unphosphorylated state [41]. Taken together, these results give support to the parameters choice in ethanol, and generate the prediction, to be tested experimentally, that Sic1 has a lower binding affinity for its cognate Cdk1-Clb5,6 complex in ethanol-grown cells compared with that of glucose-grown ones.

#### Influence of the parameters on the performance of the system: sensitivity analysis.

To test the impact of the parameter values on the dynamic behavior in glucose- and ethanol-growing cells, sensitivity analysis was performed. Classical sensitivity analysis defines as sensitivity *S* the change of a model output quantity *O* caused by the change of a parameter value *p*, such as *S* = (Δ*O/O*) / (Δ*p/p*). Since we want to study the impact of parameter changes not only on the value of a single quantity, but also on concentration time courses, we performed sensitivity analysis by calculating so-called time-dependent response coefficients *R* = (∂*c_i_* (*t*) / *c_i_* (*t*)) / (∂*p/p*) [22]. They allow tracing the—possibly varying—effect of a parameter change on a concentration during the whole simulation period. We focus on the model outputs, namely Cdk1-Cln1,2_cyt_, which drives budding (Figure S1A and S1B), and Cdk1-Clb5,6_nuc_, which drives DNA replication (Figure S1C and S1D). For example, Figure S1A indicates that a small increase of the value of parameter *k*_6_ would have the following effect on the time course of Cdk1-Cln1,2_cyt_: within the first 20 min there is almost no effect; between about 25 min and 80 min the concentration would be higher, and after 80 min there would be a small up-shift. The strongest negative response occurs with respect to *k*_5_, the rate constant of production of the inhibitor Far1. It leads to a pronouncedly lower concentration only in the period between about 35 and 75 min. Increase in *k*_49_ would decrease the concentration between 35 min to 55 min, but would increase it in the later phase. This means that increase in *k*_49_ causes a shift in the timing of the rising of Cdk1-Cln1,2_cyt_, which in turn would mean a delay in budding. Calculation of time-dependent response coefficients served to test the influence of the parameter values on the timing and strength of response. It confirmed consistency of the model structure and helped to fine-tune parameters. From the study of time-dependent response coefficients, it becomes obvious that most parameters influence the time courses of the key players of the cell cycle machinery, but to a different extent (Table S1).

### Validation of the Model

#### Response of the network to alterations in gene dosage.

**Figure 5:**
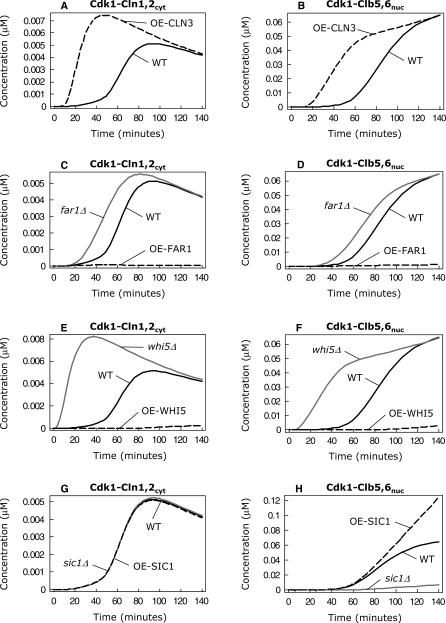
Model Predictions of Gene Dosage Effect The effects on Cdk1-Cln1,2_cyt_ (left panels) and Cdk1-Clb5,6_nuc_ levels (right panels) are compared with wild-type cells. In each panel, wild-type is shown in black, deletion mutants in gray, and overexpressed strains as a dotted line. (A,B) *CLN3* overexpression (OE-CLN3). (C,D) *FAR1* deletion (*far1*Δ) and overexpression (OE-FAR1). (E,F) *WHI5* deletion (*whi5*Δ) and overexpression (OE-WHI5). (G,H) *SIC1* deletion (*sic1*Δ) and overexpression (OE-SIC1).

To increase the stringency of the conditions in which the simulations were carried out, we investigated the effect of altering gene dosage of the major actors. Namely, we simulated deletion (0 initial level, no production) or constitutive overexpression (100-fold increase in initial level, and 100-fold increase in gene dosage). The simulated behavior of a large number of mutants is reported in Figures S2A–S2G, and summarized in Table S2. Changes in parameter values from a wild-type condition to the mutational variants are reported in Table S3. Results relating to dosage of *CLN3, FAR1, WHI5,* and *SIC1* genes, which are central to the logic of the network, are analyzed in more detail in Figure 5. The model explains the phenotype of a vast number of mutants; therefore, it appears to accurately describe the G_1_/S transition.

For instance, *CLN3* overexpression (OE-CLN3) leads to earlier overcoming of the first of the two thresholds and anticipated onset of budding and DNA replication, as experimentally observed [46,47] (Figure 5A and 5B). *far1*Δ cells show only a mild phenotype with slightly earlier entrance into S phase [15]. Strong *FAR1* overexpression, OE-FAR1, prevents budding and DNA replication effectively (Figure 5C and 5D). The huge overexpression of *FAR1* (100-fold; see above) mimics alpha factor treatment. In glucose-grown cells, average Far1 level has been reported to increase only two to three times [15]. Consistently, simulation of such a moderate overexpression only leads to a moderate delay of budding and DNA replication (see Figure S3). The *whi5*Δ mutant undergoes G_1_/S transition about 40 min earlier than wild-type. Overexpression of *WHI5* delays budding and DNA replication (Figure 5E and 5F), as experimentally observed [32,33]. The *sic1*Δ mutant should exhibit normal budding, but reduced DNA replication activity, as shown experimentally [48]. *SIC1* overexpression (OE-SIC1) is simulated to promote nuclear accumulation of Cdk1-Clb5,6, but budding takes place as in wild-type (Figure 5G and 5H), prediction that requires a new experimental test.

#### Response to various external signals.

**Figure 6:**
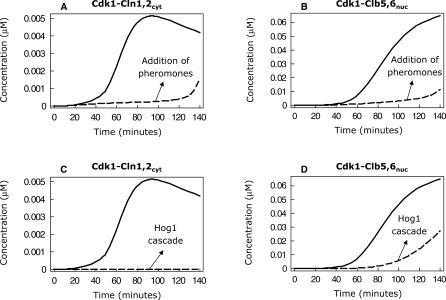
Effect of Signaling Pathway Activation on Cell Cycle Progression The effects of the pheromone pathway (A,B) and the stress-response Hog1-dependent pathway (C,D) on Cdk1-Cln1,2_cyt_ (left panels) and Cdk1-Clb5,6_nuc_ (right panels) are reported. In each panel, basal wild-type is shown in black and “activated pathways” as a dotted line.

The core of the cycle machinery has to cope with intracellular and extracellular signals that, ultimately, lead to an alteration in expression of cell cycle–regulatory proteins. Thus, we simulated the effect of signaling through the pheromone pathway and the stress-response Hog1-dependent pathway—whose modeling [21,49] lies outside the scope of this paper—by altering parameters of one or more of the biochemical reactions included in the G_1_/S transition network that are known to be modified by alterations in the signaling pathway (Figure 6 and Table S3).

The events following the pheromone pathway stimulation lead to an increase in the levels of Far1 by stabilizing the protein preventing its degradation [50]. In these conditions, the formation of a free Cdk1-Cln3_nuc_ complex is strongly reduced, thereby blocking the formation of Cdk1-Cln1,2_cyt_ and Cdk1-Clb5,6_nuc_ complexes (Figure 6A and 6B).

Hog1 pathway activation stabilizes Sic1, preventing its degradation and, concurrently, decreases the levels of Cln1,2 [51]. The major outcome is a strong decrease of Cdk1-Cln1,2_cyt_ complex formation, with a subsequent block in the appearance of Cdk1-Clb5,6_nuc_ complexes (Figure 6C and 6D). Therefore, the increase of both Far1 and Sic1 levels—when overexpressed or stabilized—results in a G_1_ arrest, as experimentally observed [50,51], supporting the involvement of both Ckis in controlling the G_1_/S transition.

### From Individual Cells to Populations in Different Growth Conditions

**Figure 7:**
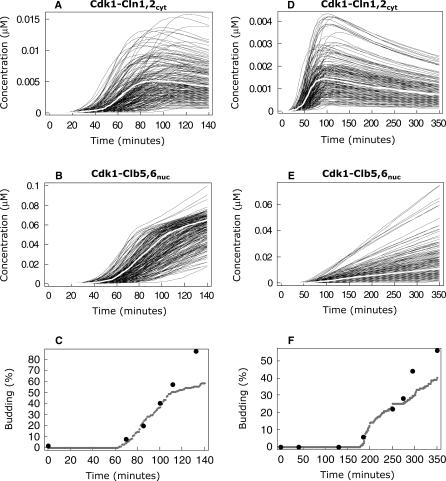
Population Effects on Budding Onset in Yeast Populations Grown in Glucose or in Ethanol To mimic differences in individual cells in a population, all parameter values are drawn from a normal distribution having the values of the ODE model as mean value. (A,D) A series of individual realizations is shown for Cdk1-Cln1,2_cyt_. The white curves represent the realizations for the original parameters. (B,E) Individual realizations for Cdk1-Clb5,6_nuc_. (C,F) Cumulative number (in %) of budded cells as a function of simulation time determined from the realizations presented in (A) and (D) (see Materials and Methods for details on calculation on budding time). Black dots refer to the experimental budding points determined for elutriated wild-type cells.

All data presented above refer to simulation of an idealized single cell. By taking into account biological variability, it is possible to simulate a cohort of synchronous cells that more closely resembles, for instance, a population of newborn elutriated cells placed to grow in a fresh medium. To this end, we modeled a population by a probabilistic approach that simulates cell-to-cell variability through repeated simulations with noisy parameters (see Materials and Methods for further details) (i.e., all parameters were sampled from a normal distribution with the original model values [Tables 2 and 3] as mean value). Figure 7 shows representative time courses for the Cdk1-Cln1,2_cyt_ and Cdk1-Clb5,6_nuc_ complexes for cells growing in glucose (Figure 7A and 7B) and in ethanol media (Figure 7D and 7E). The simulated time course of budding obtained for a population growing in glucose (black dots) is very closed to the experimentally observed one (gray curve), both in the time required for the onset of budding and in the initial slope (Figure 7C). The same simulation run for ethanol-grown cells (Figure 7F) indicates a close agreement in the timing of the onset of budding and a satisfactory slope for three subsequent points. Although the maximum value of experimental budding is not reached in both simulations, we observe a fairly good correspondence between experimental and simulated behaviors. Taken together, these data show that the model correctly predicts properties of the cells related to the G_1_ to S transition that have not been taken into account during model construction, thereby offering support to the overall consistency between input data and output performance.

### Setting of Critical Cell Size Is an Emergent Property of the G_1_/S Network

Cell viability requires the coordination between cell growth and cell division, which in budding yeast is achieved by the attainment of a nutritionally modulated critical cell size (P_S_) to trigger budding and DNA replication [2,16]. Since the present model monitors cell growth, it should be possible in principle to estimate P_S_. To do so, we need to simulate the onset of DNA replication, since operationally P_S_ is defined as the protein content of cells that enter S phase [52]. Therefore we correlate one of the outputs of the model, namely nuclear concentration of Cdk1-Clb5,6, with the onset of DNA replication. To this end, we constructed a hybrid model that uses the time course of Cdk1-Clb5,6_nuc_ as input to a probabilistic model. Given the large number of DNA replication origins present in a yeast nucleus [42,43], and the reported role of the Cdk1-Clb5,6 complex in inducing firing [39], the probability of firing for each DNA replication origin could then be related to the nuclear concentration of the Cdk1-Clb5,6 complex, as explained in more detail in Materials and Methods.

**Figure 8:**
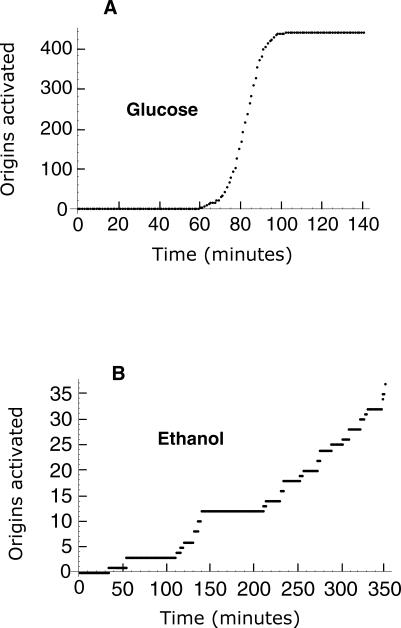
Regulation of Firing of DNA Replication Origins Cumulative number of fired origins during the course of cell cycle was calculated based on the probabilistic model for firing of origins, as explained in Materials and Methods. (A) Cells grown in glucose. (B) Cells grown in ethanol. Note different scales on the *y*-axis.

We performed simulations of the onset of DNA replication for cells grown in glucose and in ethanol media (Figure 8). In glucose (Figure 8A), activation of DNA replication origins takes place in a coordinated fashion roughly within a period between 70–90 min, in agreement with experimental data. In ethanol (Figure 8B), the Cdk1-Clb5,6 complex should be inefficiently imported into the nucleus, resulting in a longer S phase. This behavior agrees with reported data showing that a very poor carbon source such as ethanol [53], or a nitrogen source limitation, yield elongation of the S phase [54,55].

At this point, we could estimate P_S_ as the cell size when 50% of replication origins were activated in a single cell. The values of P_S_ obtained for various conditions and/or mutants, shown in Table S4 compare well with the data present in literature. In fact, overexpression of *FAR1* in glucose-grown cells results in about a doubling of Far1 level and in a modest increase in cell size and P_S_, while overexpression in ethanol-grown cells yields a larger increase in both Far1 level and cell size (P_S_) [15]. Appropriate simulations of the modulation of *FAR1* overexpression yield P_S_ values that are very consistent with our experimental data [15]. Notably, moderate overexpression of *FAR1* in a situation simulating growth in glucose results in a minor increase of P_S_ (Table S4).

**Figure 9:**
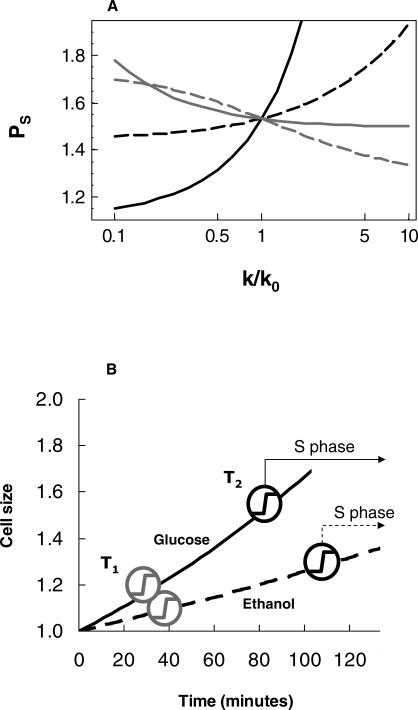
Regulation of the Critical Cell Size P_S_ Necessary To Undergo G_1_/S Transition (A) Sensitivity analysis of P_S_. For each curve, an individual parameter has been varied from 0.1-fold to 10-fold. Black line: growth rate (*k*_growth_, *k*_1_, *k*_2_ concomitantly); black dashed line: initial concentration of Far1 (far1_cyt_[0]); gray dashed line: initial concentration of Cln3 (cln3_cyt_[0]); gray line: binding value of Sic1 to the Cdk1-Clb5,6 complex (*k*_32_). (B) Simulation of the G_1_/S transition in elutriated cells indicates how the setting of P_S_ is achieved. Cells start at a volume of 1 and grow with different kinetics in glucose (solid line) and ethanol (dotted line). Overcoming of the first and second threshold, as identified by simulation, is shown by circled symbols (gray, first threshold; black, second threshold).

Having obtained a good agreement between predicted and experimental P_S_ under a set of conditions, we moved to analyze the effects of various parameters of the model on the setting of P_S_. Sensitivity analysis (Figure 9A) shows that several parameters affect its value: the initial level of Cln3 and Far1, the binding value of Sic1 to the Cdk1-Clb5,6 complex, and the growth rate. These results allow us to reconcile different, apparently conflicting, experimental results that pointed either to the increase of Cln3 concentration in the nucleus, or to the activation of transcription factors SBF/MBF, or to the regulation of Sic1 as the event setting the critical cell size. Besides, they highlight the significant role of the growth rate in determining the P_S_ value, a particularly noteworthy result that could not be obtained by visual inspection of the model reported in Figure 2.

## Discussion

We present here a detailed mathematical model of the G_1_ to S transition of the yeast cell cycle that takes into account molecular events reported in the literature and not considered in previous models, the fact that the various processes take place in both the nucleus and the cytoplasm and that components shuttle between these compartments, and the growth of the cells during the G_1_ phase. The main part of the model is formulated as a set of ODEs regarding transport and growth processes. Probabilistic modeling was included for the extensions of the model to DNA origin firing, which is instrumental in the calculation of the critical cell size required for the G_1_ to S transition and to estimation of budding.

The novelty of our model is given by several features: (1) increased accuracy of the network definition (consideration of the role of Far1 and of Whi5); (2) explicit nucleo/cytoplasmic localization; (3) the overcoming of a built-in link between cell growth and onset of S phase proposed in previous models, which is based on assumptions that are no longer tenable in the light of new experimental data (the ultrasensitive switch); (4) demonstration obtained by simulation analysis that the critical cell size is an emergent property of the entire G_1_ to S network and that it is strongly modulated by the growth rate, thereby rationalizing a wealth of literature data; and (5) prediction that the binding affinity of Sic1 to Cdk1-Clb5,6 is modulated by growth conditions, and is higher at faster growth rates.

Modeling of the cell cycle started early with mathematical models that aimed at the description of simplified systems, such as the fertilized egg, where the cell cycle is reduced to regular alternation of S phase and mitosis, with no appreciable G_1_ and G_2_ phases, and no cell growth. Such a system can well be described by an autonomous oscillatory behavior [56,57]. Subsequently, Obeyesekere and coworkers developed models for the normal mammalian cell cycle and for the G_1_ phase based on molecular interactions [58–60]. Kohn and coworkers focused on the modeling of the G_1_ to S transition for mammalian cells and proposed how the system could have evolved, beginning with the simplest functional unit, and, step by step, increased its complexity and functionality [61]. They showed that the binding of an Rb-like factor (functionally equivalent to yeast Whi5) regulates the dynamics of free E2F (functionally equivalent to yeast SBF/MBF) accumulation, and that expression of cyclin D (functionally equivalent to Cln3) and cyclin E provides a sharp trigger for the activation of E2F. They performed an intensive parameter test, similar to our approach. Various subsequent models investigate the G_1_ to S transition by focusing mainly on E2F regulation [62], and some of them studied the dynamics using bifurcation analysis [63–65].

The most relevant molecular model describing the budding yeast cell cycle has been proposed by the Tyson group [19,20]. It has been iteratively refined and amended during the years, and most attention in recent years has gone towards improving the description of the mitotic network [19]. A more recent paper from the same group [66] argued that different facets of cell cycle regulation in different organisms might be accounted for as variations of a “generic” cell cycle model that includes a number of modules describing the various cell cycle aspects.

The model presented in this paper represents a molecular blow-up of the G_1_/S transition in budding yeast, and at this stage does not include a description of other cell cycle events. As is customary in modular systems biology, working on a single module or subsystem allows us to increase the number of components considered in the network, and to sharpen the focus on the wiring of the various components of the subsystem at hand, while leaving open the possibility of linking the module to other modules through its input and outputs.

The major mechanistic differences that set apart the network described and tested by simulation in the present paper from the G_1_ to S transition subsystem of the Tyson model [19,20] are summarized and briefly discussed below.

### Involvement of Far1 and Cln3 in the First, Growth-Sensitive Threshold

First of all, our model considers that the control over the entrance into S phase is distributed over two sequential thresholds that involve Cdk1, cyclins, and two distinct inhibitors: the Ckis Far1, in the first threshold, and Sic1, which acts on the second threshold. While Sic1 has long been recognized as regulating initiation of DNA replication, the involvement of Far1 in the control of the Cdk1-Cln3 activity is not present in earlier models [19,20]. A role for an inhibitory molecule (either a Cki or a phosphatase) in setting of P_S_ has been proposed independently by different groups [10,14]. Such an inhibitor was expected to present a peak in late M phase, a basal, low synthesis during the other phases of the cycle, and have a Cdk1-Cln–dependent degradation [10]. This pattern, initially suggested for Sic1, fits well also for Far1 [31,67].

Since inhibition of Cln3-Cdk1 by Far1 is a major innovative feature of the network proposed in this paper, let us briefly review relevant experimental evidence about this item in addition to that reported in the section describing the construction of the model, regarding physical interaction, biochemical activity, and genetic interaction. Direct identification of the Cdk1-Cln3-Far1 complex in mitotic cells has not yet been reported. The lack of genome-wide two-hybrid or mass spectrometry data to support the interaction of Far1 and Cdk1-Cln3 is not surprising, since the high unreliability of these data is well-known. In fact, comparison between two-hybrid datasets (obtained independently from two laboratories) and mass spectrometry data (also obtained by two different groups) showed a surprisingly small overlap: about 10% and 14%, respectively (as reviewed by Ito et al. [68]). Moreover, false signals have been estimated to be as high as 50% [69]. On the other hand, in α-factor–treated cells, coimmunoprecipitation experiments showed that Far1 became a sizable co-precipitated substrate of Cdk1-Cln3 [70], and biochemical assays indicate that Far1 inhibits the activity of Cdk1-Cln3 in immunoprecipitates after α-factor treatment [71]. Since activation of Fus3 protein kinase determined by pheromone treatment promotes increased interaction of Far1 to Cdk1-Cln3, it would be reasonable to hypothesize that a basal binding between Cdk1-Cln3 and Far1 is present also in the absence of α-factor. It must be stressed that neither the report of Tyers and Futcher [70] nor that of Jeoung et al. [71] were designed to address Cdk1-Cln3-Far1 interaction in mitotic cells, but only in the presence of pheromone.

More compelling experimental evidence for the involvement of Far1 in the G_1_/S transition of mitotic cycles has recently been accumulated. By analyzing mutants in *CDC48,* a cell cycle gene essential in the protein degradation pathway that acts as an ubiquitin-selective chaperone, Fu et al. [29] showed that blocking of Far1 degradation results in cell cycle arrest in G_1_ in mitotic cells. Alberghina et al. [15] independently showed that both *FAR1* deletion and its overexpression affect the critical cell size P_S_ as expected for a Cln3 inhibitor, and that deletion of either *CLN3* or *FAR1* in a *sic1*Δ background has an almost identical effect on nutritional modulation of cell size and P_S_. These findings are consistent with the notion that Far1 is acting in the same pathway as Cln3 with regard to nutritional modulation of cell size.

**Figure 10:**
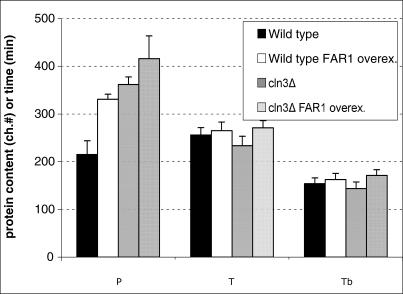
Control of Cell Size by Far1 Acts through Cln3 Protein content (P; [i.e., the average protein content determined using flow cytometry of FITC-stained cells]), doubling time (T), and length of the budded phase (Tb) for wild-type (black bar, control; white bar, *FAR1* overexpression) and *cln3**Δ*** strain (dashed bar, normal; checkered bar, *FAR1* overexpression). Protein content is expressed as arbitrary units (i.e., channel number determined from FACS analysis), T and Tb in minutes.

To prove more formally that Far1 is acting on Cln3 and not on the closely related Cln1,2 cyclins, the effect of Far1 overexpression on the cell size of *cln3*Δ cells in exponential growth on ethanol-supplemented medium—chosen because these are the growth conditions in which the effect of Far1 overexpression on cell size is larger [15]—was tested and compared with that of wild-type cells. Figure 10 shows that, as previously reported [15], in wild-type cells overexpression of *FAR1* leads to a large increase in cell size (66%). Such an increase is highly statistically significant (Student *t*-test, *p* < 0.01). The increase in cell size is much lower (∼15%) in cells devoid of the *CLN3* gene. Such an increase in cell size is at the borderline of statistical significance (Student *t*-test, *p* = 0.09). Put in another way, ∼80% of the increase in size brought about by Far1 overexpression is lost in the absence of the *CLN3* gene. These data appear even more relevant when one takes into account that the amount of Cln3 in growing yeast cells (which, according to these newly presented data, contributes to the majority—and possibly all—of the effect of Far1 on cell size) is much lower than the amount of Cln1,2 protein [24–27].

Taken together, literature results independently obtained by several laboratories and experimental findings newly presented here indicate that the assumption made in our model (i.e., that Far1 plays a role in the control of entrance into S phase of mitotic cells by inhibiting Cdk1-Cln3), has a firm base, although our understanding of its mode of action is not yet complete.

The effect that the introduction of the Cln3/Far1 threshold has on cell cycle dynamics must be stressed: it allows the cells to detect the reaching of a given size (set by the amount of Far1 present in a cell) when the amount of Cln3, which increases in proportion to cell mass, overcomes Far1. The actual critical cell size is reached when the cells overcome the second threshold dependent on the Cki Sic1.

### Modeling Nucleo/Cytoplasmic Compartmentalization

Recent evidence showed that alterations in nucleo/cytoplasmic localization of cyclins [28] and Ckis [17] play a relevant regulatory role in cell cycle progression. Nuclear localization of Cdk1-Cln3 and Cdk1-Clb5 complexes is assumed throughout recent papers by Tyson and coworkers [19,66], but is in fact modeled in a simplified way by multiplying cyclin synthesis rates with the parameter mass. This implementation gives different patterns of accumulation to cyclins as compared with other components. However, compartmentalization involves much more than that, since: (1) proteins are synthesized in the cytoplasm, starting from mRNA migration from the nucleus; (2) both import and export can be independently regulated; and (3) controlled partitioning affects binding equilibria by altering the actual concentration of a given protein available for binding to a given interactor within a subcellular compartment. None of these regulatory issues is addressed in the models of Chen et al. [19,20]. On the contrary, shuttling of proteins and complexes in and out of the nucleus is a major, explicitly modeled feature of our network that allows us to address biological significance of subcompartmentalization of biochemical reactions, avoiding inconsistencies intrinsically present in the Chen et al. model, where “nuclear” Clb proteins are free to interact with “cytoplasmic” Sic1, thus violating a major biochemical consequence of subcellular compartmentalization.

It is relevant at this point to mention that it is the explicit modeling of nuclear compartmentalization that allows us to make predictions regarding biochemical properties of the Cdk1-Clb5,6-Sic1 complexes (discussed in the Results section), which have to be verified experimentally in the iterative procedure characteristic of systems biology [72–74]. The suggestion that comes from our simulation analysis indicates that Sic1 should have a higher binding affinity for the complex Cdk1-Clb5,6 in cells grown in glucose in comparison with cells grown in ethanol, and therefore stresses the interest in analyzing the phospho-signature of Sic1 in two conditions. Although we do not want at this stage to be bound to any specific hypothesis, it is also worth remembering that we have shown that a Sic1-derived peptide has a dramatically increased affinity towards a heterologous Cdk-cyclin complex in the CK2-phosphorylated versus the unphosphorylated state [41], as CK2 is a protein kinase whose activity is found to be higher in fast-proliferating cells [75].

### Coupling Cdk1-Cln3 Activity to the Downstream Events

The trigger that controls the activation of transcription factors SBF/MBF in Chen et al. [19,20] is modeled according to a zero-order ultrasensitivity switch [76,77], a function that was chosen because it is suitable for the phosphorylation/dephosphorylation reaction assumed to control SBF/MBF activation [19,20]. It was assumed that each transcription factor (both SBF and MBF) exists either in an active or inactive form, and that the transition between the two forms is catalyzed by two competing enzymes: a protein kinase (Cdk1-Cln3) and a protein phosphatase (whose existence was only supposed at the time of the construction of the model), each of which follows Michaelis–Menten kinetics. The transitions are taken to be fast enough to maintain each transcription factor in a steady-state distribution. The kinetic equation describing the behavior of the Goldbeter and Koshland ultrasensitive switch [56] assumes a sharply sigmoidal function when the cell grows through the critical size. The value of cell mass that triggers the SBF/MBF switch has been calculated from a quadratic equation containing half a dozen variables, and the solution has been set to 1.2 (1 being the cell mass of the newborn cell), the large margin of error being the result of a determination that requires the estimation of a sizable number of kinetic and efficiency parameters.

Subsequent experimental work has shown that the activation of SBF/MBF is not due to direct phosphorylation of transcription factors, with a delicate equilibrium between a kinase (Cdk1-Cln3) and a phosphatase (never found) as assumed by the very basic assumptions of the Chen et al. model [20]. Instead, as we modeled, the activation of SBF/MBF is due to the dissociation of an inhibitor (Whi5) that is phosphorylated by Cdk1-Cln3 [32]. The interest of this regulatory link is further stressed by the fact that Whi5 has the same role as the retinoblastoma protein in the control of cell cycle progression in mammalian cells [33].

The predictive ability of the Chen et al. [20] model is due to the built-in control for the onset of S phase given by the estimation of the critical cell size at 1.2 that fortunately is very close to the experimental one for cells growing in glucose. In fact, it has been common knowledge for many years, derived from quantitative microscopic observations, that cells growing in batch in glucose medium at the onset of budding are only slightly larger than newborn daughter cells [78,79]. A double-tag flow cytometric analysis performed in one of our laboratories on chemostat-grown cells at variable glucose dilution rates has clarified that the ratio of critical cell size at the onset of S phase/cell size of newborn daughters is about 1.5 at low-growth rates, then decreasing to 1.24 at fast-growth rates [52]. Therefore, the value 1.2 is quite close to the actual one if we consider cell growing in glucose, but it should not be used to simulate cell cycles at low-growth rates.

Based on recent biochemical and genetic evidence [15,29], here we show that a simpler, better-defined mechanism (i.e., the Cln3/Far1 threshold) brings about a switch-like accumulation of nuclear SBF and MBF transcription factors not at a prefixed cell mass, but as a result of the dynamics of the regulatory molecules (see Figure 3B), thus effectively coupling cell growth to the onset of DNA replication and budding. The ensuing Cdk1-Cln3 activity phosphorylates Whi5 [32,33], and leads to the activation of transcription factors SBF/MBF, opening up the pathway that leads to the onset of DNA replication.

### The Critical Cell Size Is an Emergent Property of the G_1_ to S Network

The requirement of a critical cell size to enter into S phase has been known for several decades, but its molecular basis is still under discussion. Cln3 is certainly involved in the setting of the critical cell size, but does not work alone in the mechanism. In fact, while cells growing at faster growth rates are larger than those growing at slower rates [12], in any given medium, cells overexpressing Cln3 are smaller and have a shorter G_1_ phase than wild-type cells [2,9], a paradox pointed out by Heideman and collaborators [11]. Results of the sensitivity analysis summarized in Figure 9A neatly show that P_S_ does not originate from the properties of a single molecule, but rather is an emergent property of the network structure. Besides the Cln3 and Far1 dosage, the growth rate is a major factor in setting P_S_. The role of the growth rate in determining the value of P_S_ can be explained as follows. The value of the cell size at the traverse of the first cell sizer threshold is quite similar both in rich and in poor carbon sources (Figure 9B), since the Cln3/Far1 ratio remains almost equimolecular at the various growth rates [15], with both Cln3 [11] and Far1 [15] increasing at faster growth rates. As shown before, a sizable period of time is needed to move from the first to the second threshold both in glucose and in ethanol. Since it is the traverse of the second threshold that actually sets P_S_, its value clearly will be much larger in cells that grow faster (Figure 9B).

We use the term “emergent property” with true systems biology significance: a property that individual components of the G_1_ to S network do not have but that emerges from their interaction [80]. Of course, since the setting of P_S_ is an emergent property of the entire G_1_ to S network, other parameters—such as the accumulation rate of Cln3 and/or Far1, or the binding affinity of Sic1 to the Cdk-cyclin complexes—affect P_S_ independently from the growth rate and may become relevant in appropriate growth conditions.

In the current Tyson model(s), the onset of DNA replication is controlled by a single event: a cell sizer is taken to be operative only at low-growth rates, while an oscillator mechanism is assumed to be active at fast-growth rates [66]. In our model, a sizer mechanism is operative at all growth rates, and the presence of two distinct—temporally spaced—thresholds acting together to set P_S_ not only introduces a delay, but also makes the delay sensitive to the growth rate (Figure 9B).

Taken together, the results presented in this paper offer an example of the usefulness of a systems biology approach for the understanding of complex biological processes.

## Materials and Methods

### Deterministic model for concentration changes.

The model comprises equations for production and degradation of mRNA and proteins and for the formation of dimeric and trimeric protein complexes (Figure 2). It accounts for the nucleo/cytoplasmic localization of the compounds. Transport processes are described like reactions, converting, for example, Cdk1_cyt_ into Cdk1_nuc_. Cell growth is characterized as exponential increase in volume. All concentration changes are dependent on the volume changes of the respective compartment. The full set of equations and parameters can be found in Tables 1–5, and the impact of the chosen parameters on behavior of the simulated system was studied using sensitivity analysis [22].

We explicitly consider two compartments, the nucleus and the cytoplasm, with volumes *V_nuc_* and *V_cyt_,* respectively. Nuclear and cytoplasmic compounds are denoted by the subscripts *nuc* or *cyt,* respectively. The dynamics of the concentration of every compound is determined by three different types of processes: (1) biochemical reactions, (2) transport over the nuclear membrane, and (3) change of the volumes. The dynamics are described by sets of ODEs. The temporal evolution of a nuclear compound reads

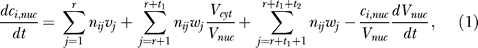
and the temporal evolution of a cytoplasmic compound is given by

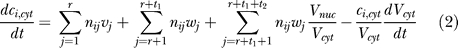
for *i* = 1,..,*m*, where *m* is the number of biochemical species with the concentrations *c_i_* (either nuclear or cytoplasmic). The quantity *r* is the number of biochemical reactions with the rates *v_j_*, and *t*_1_ and *t*_2_ are the number of transport steps from cytoplasm to nucleus and vice versa with the rates *w_j_*. The quantities *n_ij_* denote the stoichiometric coefficients of the compounds in the respective reactions or transport steps. The volume changes are given by

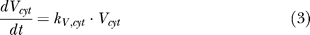
and

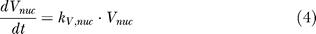
where *k_V,cyt_* and *k_V,nuc_* are the rate constants of volume change.

All individual reaction rates *v_j_* and transport rates *w_j_* are governed by irreversible mass action kinetics:

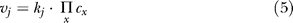
and

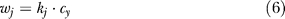
where *k_j_* are the rate constants and the index *x* runs over all substrates and modifiers of reaction *j*, and the index *y* denotes the compound transported in step *j*.

### Estimation of parameter values.

*Rate constants for production.* The model comprises 54 rate constants. The only relevant transcriptional control implemented in the model relates to transcription of *CLN1,2* and *CLB5,6* genes, which is dependent on SBF/MBF. The rate for *CLB5,6* transcription (*k*_2_) was estimated from kinetics of Clb5 production in elutriated cells (this article). The same value was used for *CLN1,2* transcription (*k*_1_). The rates of translation (*k*_4_ and *k*_3_, respectively) were set one order of magnitude higher. Rate constants for production of Far1 (*k*_5_) and Cln3 (*k*_6_) were estimated from kinetics of production of the appropriate protein in elutriated cells growing in glucose and in ethanol medium [15]. Basal level of production of Whi5 (*k*_8_) and Sic1 (*k*_9_) was set the same as *k*_5_. Since the Cdk1 level is never limiting, the rate constant for Cdk1 production (*k*_7_) was set much higher than those for Cdk-regulatory proteins. The Cln3-independent production of SBF/MBF (*k*_35_), implemented to mimic the contribution of Bck2, was set low.

*Rate constants for degradation.* In the absence of other relevant information, rate constants for degradation of proteins were set to the same value, 0.01 min^−1^. Rate constants for degradation of mRNAs were set one order of magnitude higher. The rate constants for degradation of Cln2 and Clb5, *k*_12_ and *k*_13_, respectively, were derived from previous cell cycle models [19,20]. The rate constant for degradation of cytoplasmic Cdk1 was derived assuming a steady partitioning of Cdk1 between nucleus and cytoplasm, while degradation of nuclear Cdk1 (*k*_21_) was considered negligible and set to 0.

*Rate constants for association and dissociation of protein complexes.* Values of association and dissociation constants for proteins involved in Cdk-cyclin-Cki complexes formation were not available. Rate constants derived from BIAcore (http://www.biacore.com) data regarding interaction between Sic1 and heterologous Cdk-cyclin [40,41] were thus used, assuming that regardless of the actual Cdk, cyclin, or Cki involved, relative scale of interaction affinity should be conserved. The same association value was then used for the association constant between SBF and Whi5 (*k*_34_). These values guided us also in choosing the value for dissociation of Cdk-cyclin complexes (*k*_25_, *k*_27_, *k*_29_) and of these complexes containing Cdk inhibitors (*k*_31_, *k*_33_).

*Rate constants for phosphorylation reactions.* Michaelis–Menten kinetics for phosphorylation reactions were simplified to mass action (i.e., substrate concentration was considered low compared with *K*_m_). Such simplification did not significantly affect simulation results (unpublished data). The resulting constants are thus near to the *k*_cat_/*K*_m_ ratio. Data for *k*_cat_ and *K*_m_ for typical Cdk-catalyzed phosphorylation reactions [81] were thus used.

*Rate constants for transport between nucleus and cytoplasm.* In the absence of more specific experimental information regarding “in vivo” constants, rate constants for nuclear import of single proteins (Far1, Cln3, Cdk1, and Whi5) were set equal (*k*_42_–*k*_45_). The rate constants for nuclear export were then set to favor nuclear localization. Similarly, nuclear localization was favored for the Cdk1-Cln1,2 binary complex. According to experimental data indicating that Sic1 promotes Clb5 nuclear localization [17], nuclear import of the ternary complex Cdk1-Clb5,6-Sic1 (*k*_47_) was favored over transport of the cognate binary complex (*k*_48_). Rate constants for nuclear export of *CLN1,2* and *CLB5,6* mRNAs (*k*_50_ and *k*_51_, respectively) were set larger than those of corresponding proteins.

*Rate constants of exponential growth.* Values for glucose- and ethanol-grown cells were obtained by averaging literature data [15] and unpublished data from our laboratory.

*Absolute and relative initial concentration of protein and protein complexes.* The average number of molecules in glucose-grown cells was taken from Ghaemmaghami et al. [25], which was related to the maximal values of that compound assumed during time course. The Far1/Cln3 ratio in newborn cells was taken from Alberghina et al. [15].

*Sensitivity analysis.* Sensitivity analysis was performed to test the influence of the parameter choice on the systems dynamics (Figure S1). To this end, we calculated the time-dependent response coefficients [22], defined as

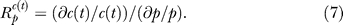



These coefficients indicate the direction and amount of change of the time course for the concentration *c*(*t*) upon an infinitesimal change of the parameter (or initial concentration) *p*. Loosely spoken, one can also interpret this as the percentage change of the concentration over time upon a 1% change of the parameter. During model development, the response coefficients were used to indicate appropriate parameter changes, since there are not enough data available to estimate the parameters by a global approach.

*Probabilistic model for firing of the DNA replication origins.* The influence of Cdk1-Clb5,6_nuc_ on the licensing of replication origins and DNA replication is described by a probabilistic three-step model that does not regard molecular details of this highly complex process. Step 1 lumps all events from free origin to pre-replicative complex. The transition time for each of the 440 replication origins is taken from a normal distribution with mean of 15 min and standard deviation of 2 min. Step 2 is Cdk1-Clb5,6_nuc_ dependent. The probability for performing Step 2 at a certain time is determined by the concentration of Cdk1-Clb5,6_nuc_ at that time. The period of this step is necessary for Cdk1-Clb5,6 to exceed a value taken from a normal distribution with a mean of 0.03 μM and standard deviation of 0.01 μM. The transition time for Step 3 to reach the “fired” state is again taken from a normal distribution with a mean of 1 min and standard deviation of 0.01 min. When the origin has fired, then DNA replication proceeds bidirectionally from multiple replication origins, as experimentally reported [82,83]. If replication reaches the neighboring origin before it fires on its own, that origin is set to the state “fired.” The distance between DNA replication origins is fixed.

We considered that in ethanol-growing cells—with a growth rate about 2-fold lower compared with the glucose-growing cells—the fork rate is about one-half (and the time of origins activation is doubling) than the glucose ones. This assumption agrees with the reported data, in which the longer S phase in yeast cells growing in poor nitrogen medium can be accounted for by a reduction in replication fork rate [55].

*Probabilistic model for budding.* Assuming that the parameters for the individual cells may vary around their mean values as given in the parameter list in Tables 2–5 (standard deviation of *k*_i_*0.287), we can simulate the time courses of Cdk1-Cln1,2_cyt_ for cell populations. Onset of budding is determined by the time *t_max_*_/2_ when Cdk1-Cln1,2_cyt_ reaches a critical value equal to the half-maximal concentration obtained for mean parameter values. As time for the appearance of the bud, we use *t_budding_* = *t_max/2_* (*Cdk1Cln1,2_cyt_*) + *t_delay_*, with *t_delay_* = 0 min in glucose and *t_delay_* = 120 min in ethanol.

*Yeast strains, cell growth, and cell size determination.* Yeast cells W303-CF (*cln3::KAN1, pCLN*3–15Myc, *FAR*1–15Myc-*URA3*), FAR1^tet^ (*cln3::KAN1, pCLN*3–15Myc, *far1::HIS3,* pTet-*FAR*1–15Myc), and *cln*3Δ (*cln3::KAN1*) were grown in synthetic complete media supplemented with ethanol as a carbon source. Growth conditions, media, and flow cytometry determinations were conducted as described previously [15].

*Simulation tool.* Numerical calculations have been carried out using Mathematica Version 5.1 (Wolfram Research, http://www.wolfram.com).

## Supporting Information

Figure S1 Sensitivity Analysis of the G_1_ to S Network The analysis in the form of time-dependent response coefficients is used to estimate the influence of the choice of rate constants and initial concentrations on the timing and strength of response. Each curve represents the relative change of a concentration time course *c*[*t*] caused by an infinitesimal increase in the denoted parameter *k* (∂*c*[*t*]*/* ∂k*)*).
(A–B) Time-dependent response coefficients for Cdk1-Cln1,2_cyt_, which supports budding.
(C–D) Time-dependent response coefficients for Cdk1-Clb5,6_nuc_, which supports DNA replication.
(65 KB PDF)

Figure S2 Effect of Single and Multiple Alterations in Gene Dosage on the Network Outputs (A–G) The results on Cdk1-Cln1,2_cyt_ (left column) and on Cdk1-Clb5,6_nuc_ (right column) are reported.
(79 KB PDF)

Figure S3 Effect of *FAR1* Overexpression on the Network Outputs The results on Cdk1-Cln1,2_cyt_ and for Cdk1-Clb5,6_nuc_ are reported for glucose-growing cells (A) and ethanol-growing cells (B).
(32 KB PDF)

Table S1 Parameters That Influence the Sensitivity Analysis Response Positive or negative responses of rate constants and initial concentrations on the time courses of Cdk1-Cln1,2_cyt_ (drives budding) and Cdk1-Clb5,6_nuc_ (drives DNA replication) complexes.
(42 KB PDF)

Table S2 Effect of Mutants or Genetic Modifications on G_1_/S Transition in Yeast Cell Cycle Results from model simulation and experimental results from the literature are reported.
(56 KB PDF)

Table S3 Parameters Used for the Mutational Variants The values are referred to the simulations reported in Figure S2, and which phenotype is described in Table S2.
(77 KB PDF)

Table S4 Estimated P_S_ Values The critical cell size is calculated for wild-type cells grown in glucose and in ethanol media, and for deletion and overexpression of mutational variants of the model. If no value is given, P_S_ was not reached within the simulation time of 140 min.
To give a more satisfactory profile for the *cln3*Δ strain—to agree with reported data [15]—it is possible to obtain a reasonable P_S_ value increasing the values of two parameters (i.e., the Cln3-independent synthesis of *CLN1,2* and *CLB5,6* [*k*_35_] and the Sic1-independent transport of Cdk1-Clb5,6 [*k*_48_]).
(36 KB PDF)

## Abbreviations

Cdk: cyclin-dependent kinase
Cki: Cdk inhibitor
ODE: ordinary differential equation
