# Supplementary material for: Cell Size at S Phase Initiation: An Emergent Property of the G1/S Network
Source: PLoS Comput Biol. 2007 Apr 13;3(4):e64. doi: 10.1371/journal.pcbi.0030064 (PMC1851985; doi:10.1371/journal.pcbi.0030064)
Supplement: Supplementary file 1 [file pcbi.0030064.sg001.pdf]

# Figure S1A

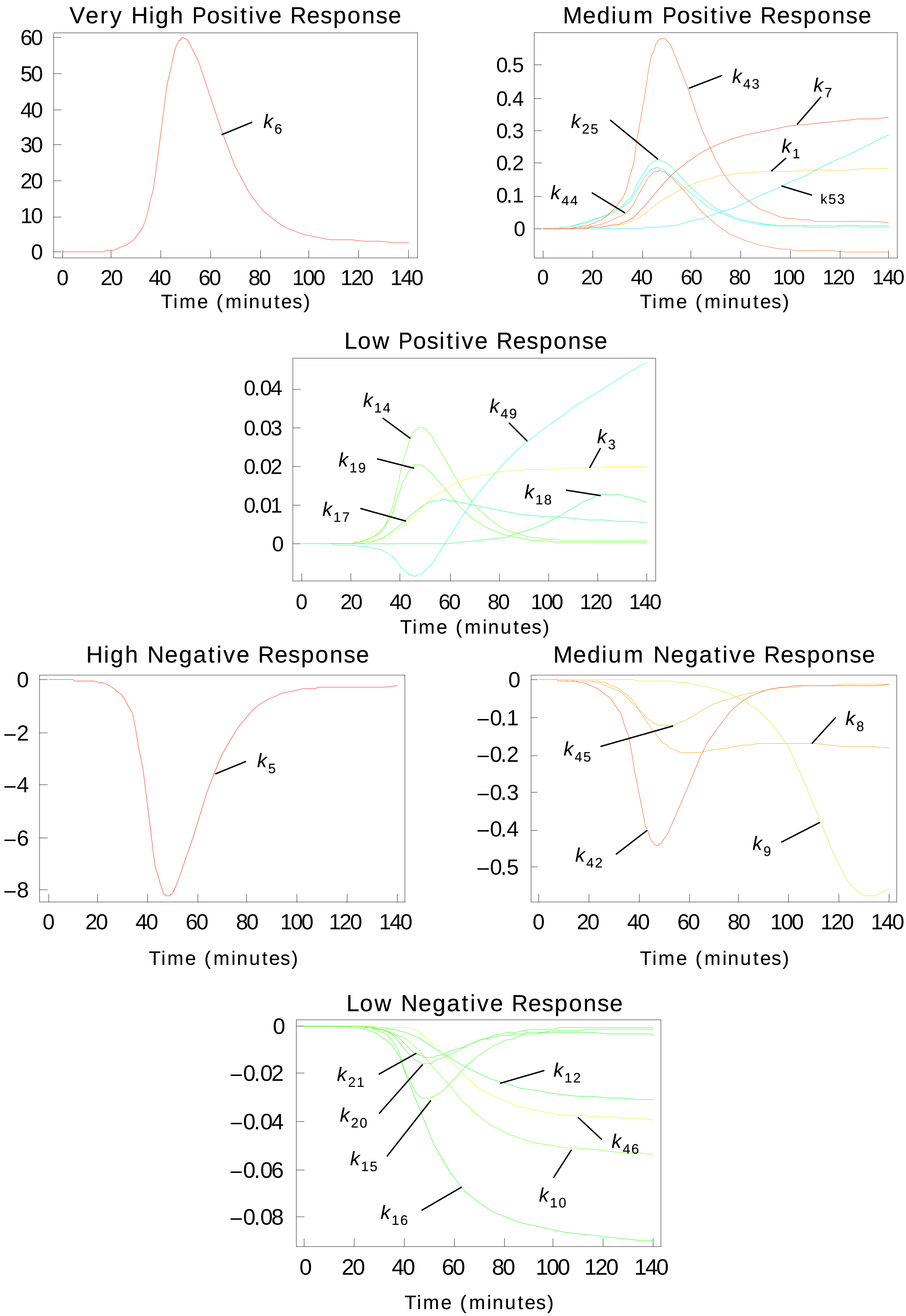

Very High Positive Response
$k_6$
Time (minutes)
Medium Positive Response
$k_{43}$
$k_7$
$k_{25}$
$k_1$
$k_{44}$
k53
Time (minutes)
Low Positive Response
$k_{14}$
$k_{49}$
$k_3$
$k_{19}$
$k_{18}$
$k_{17}$
Time (minutes)
High Negative Response
$k_5$
Time (minutes)
Medium Negative Response
$k_8$
$k_{45}$
$k_{42}$
$k_9$
Time (minutes)
Low Negative Response
$k_{21}$
$k_{12}$
$k_{20}$
$k_{46}$
$k_{15}$
$k_{10}$
$k_{16}$
Time (minutes)

# Figure S1B

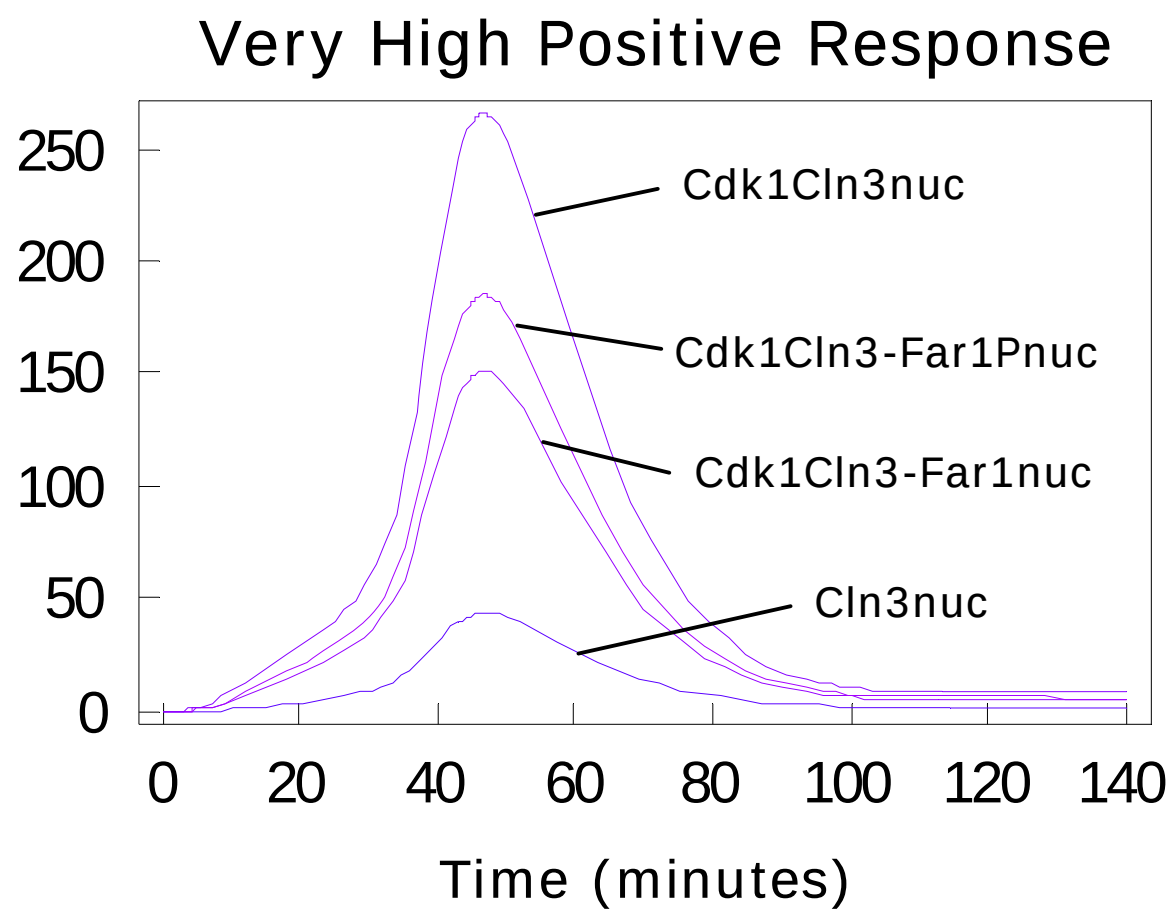

Very High Positive Response
Cdk1Cln3nuc
Cdk1Cln3-Far1Pnuc
Cdk1Cln3-Far1nuc
Cln3nuc
250
200
150
100
50
0
0
20
40
60
80
100
120
140
Time (minutes)


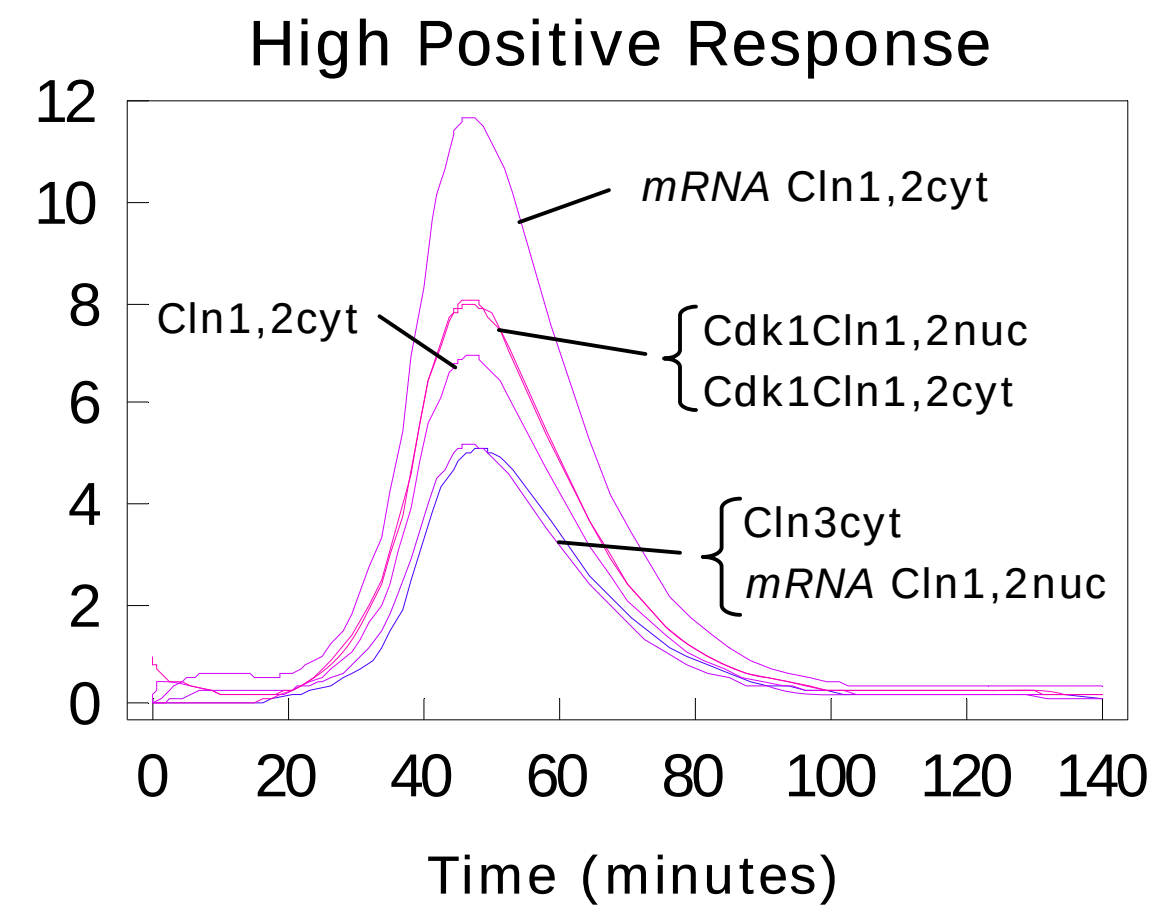

High Positive Response
mRNA Cln1,2cyt
Cln1,2cyt
Cdk1Cln1,2nuc
Cdk1Cln1,2cyt
Cln3cyt
mRNA Cln1,2nuc
12
10
8
6
4
2
0
0
20
40
60
80
100
120
140
Time (minutes)


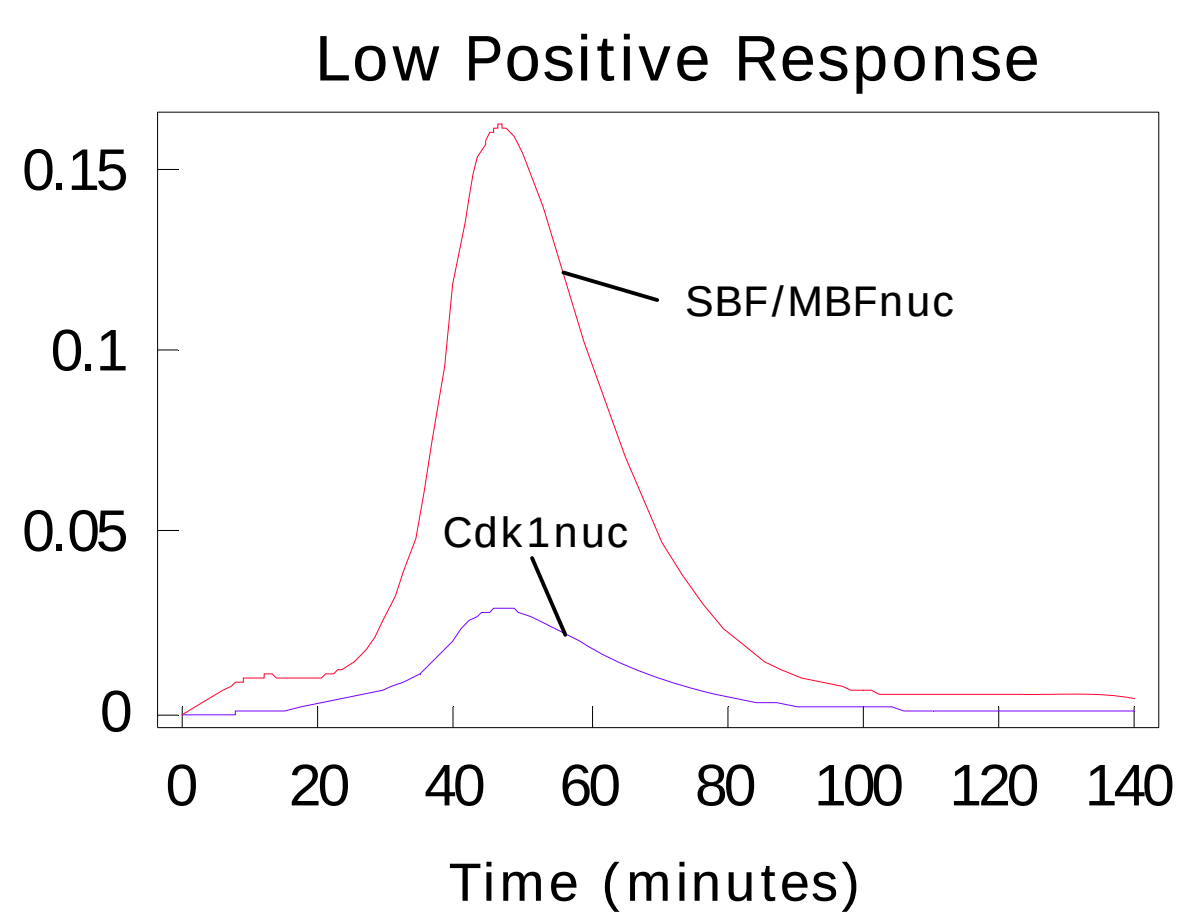

Low Positive Response
SBF/MBFnuc
Cdk1nuc
0.15
0.1
0.05
0
0
20
40
60
80
100
120
140
Time (minutes)


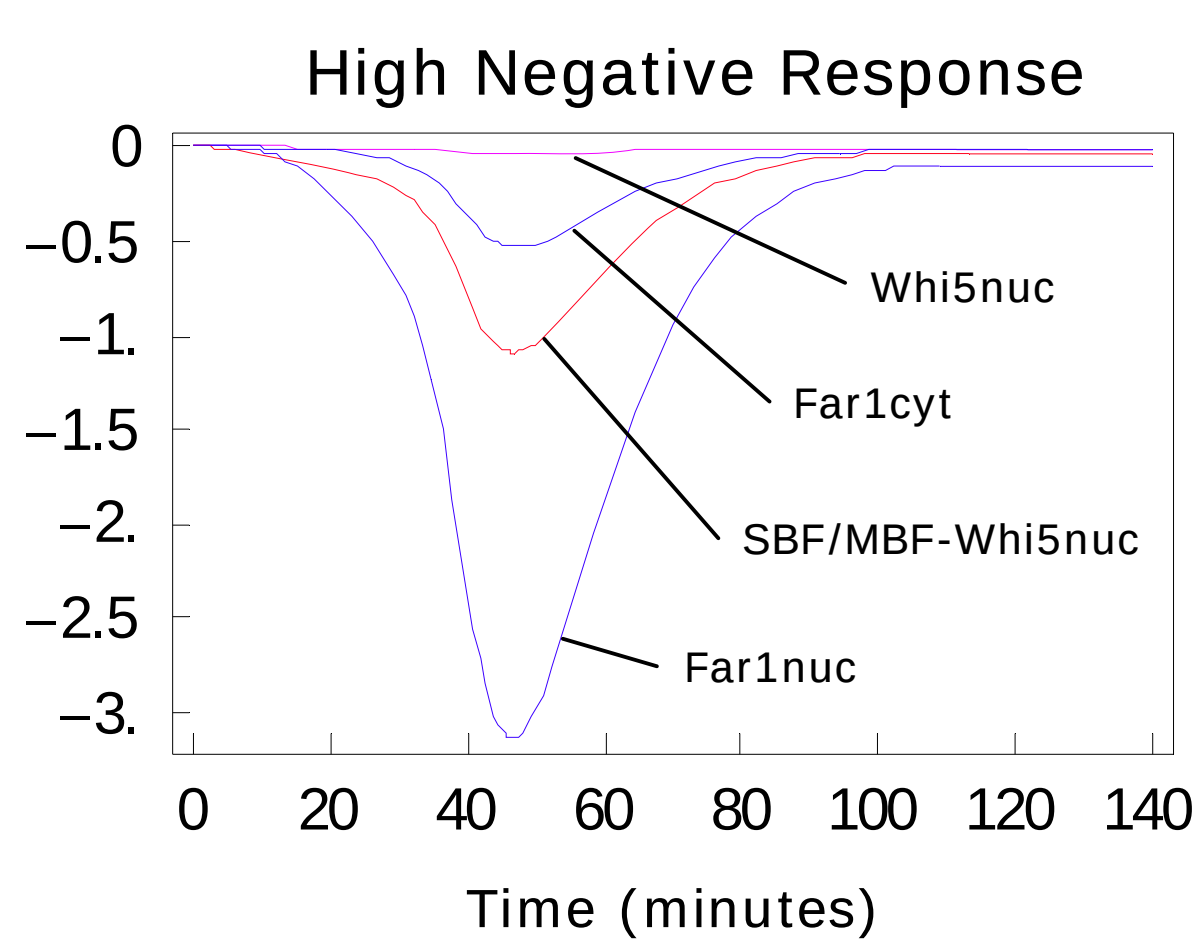

High Negative Response
Whi5nuc
Far1cyt
SBF/MBF-Whi5nuc
Far1nuc
0
−0.5
−1.
−1.5
−2.
−2.5
−3.
0
20
40
60
80
100
120
140
Time (minutes)

# Figure S1C

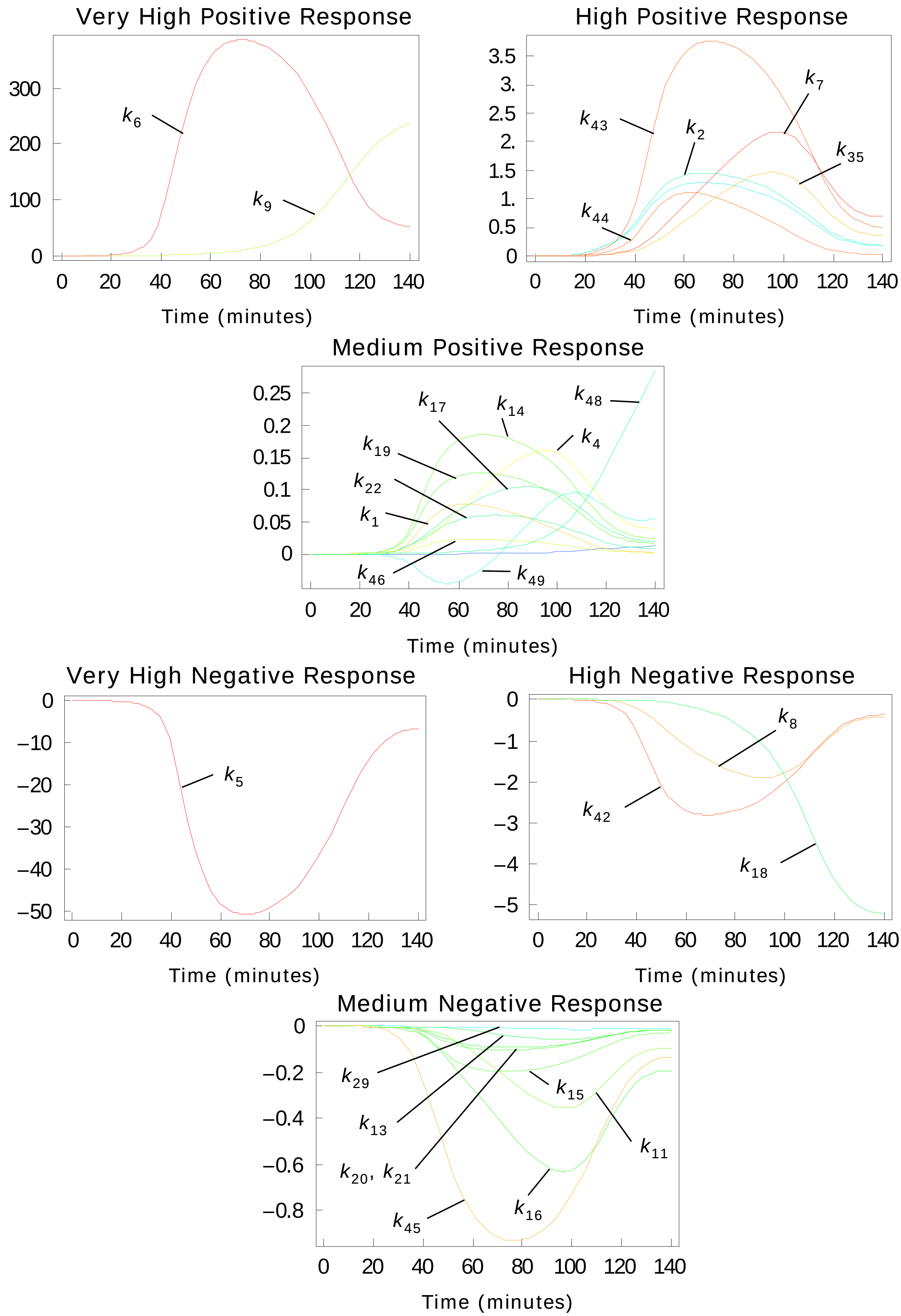
Very High Positive Response
300
200
100
0
k6
k9
0 20 40 60 80 100 120 140
Time (minutes)
High Positive Response
3.5
3.
2.5
2.
1.5
1.
0.5
0
k43
k7
k2
k35
k44
0 20 40 60 80 100 120 140
Time (minutes)
Medium Positive Response
0.25
0.2
0.15
0.1
0.05
0
k17
k14
k48
k19
k4
k22
k1
k46
k49
0 20 40 60 80 100 120 140
Time (minutes)
Very High Negative Response
0
−10
−20
−30
−40
−50
k5
0 20 40 60 80 100 120 140
Time (minutes)
High Negative Response
0
−1
−2
−3
−4
−5
k8
k42
k18
0 20 40 60 80 100 120 140
Time (minutes)
Medium Negative Response
0
−0.2
−0.4
−0.6
−0.8
k29
k15
k13
k11
k20, k21
k16
k45
0 20 40 60 80 100 120 140
Time (minutes)

# Figure S1D

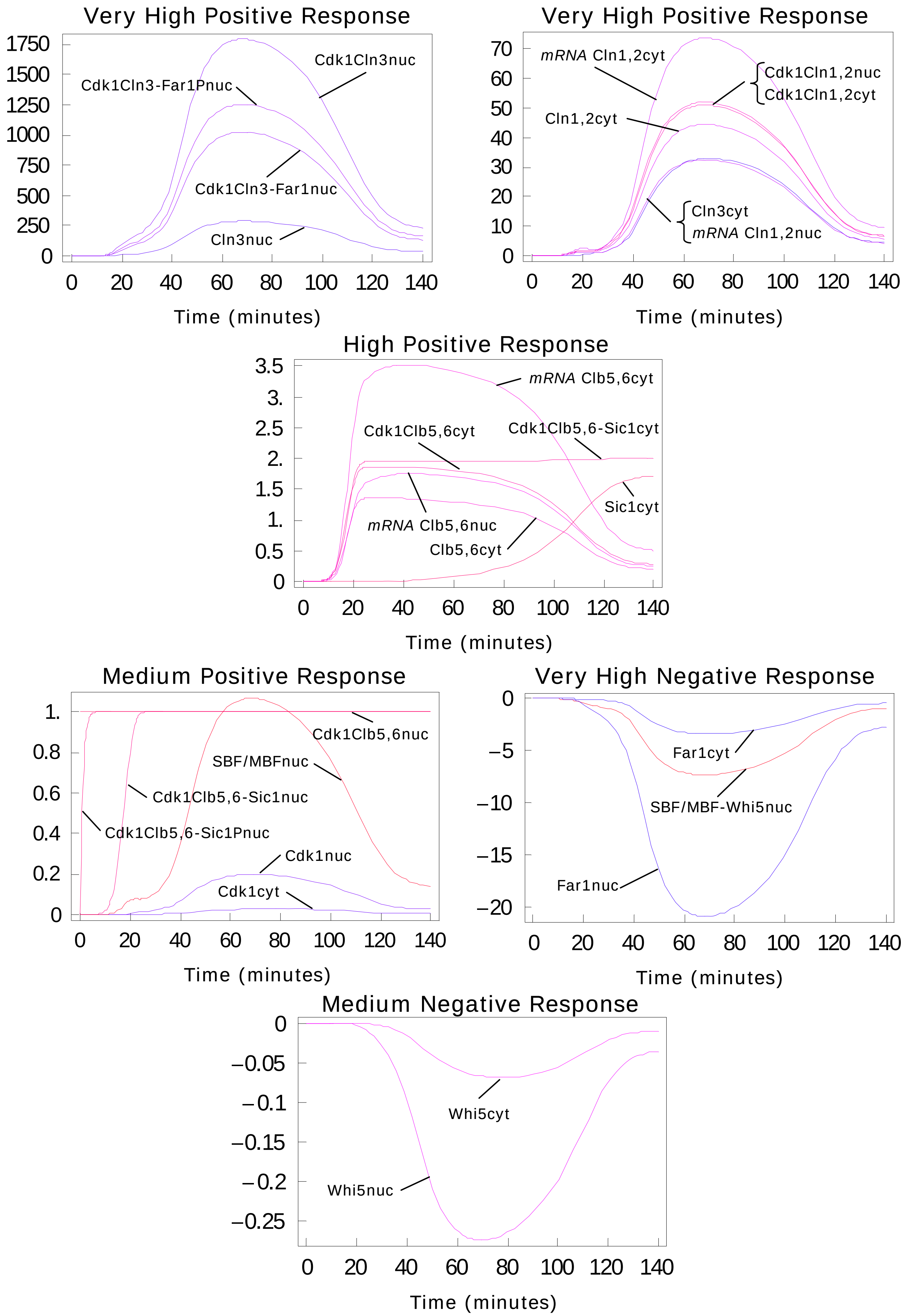
Very High Positive Response
Cdk1Cln3nuc
Cdk1Cln3-Far1Pnuc
Cdk1Cln3-Far1nuc
Cln3nuc
1750
1500
1250
1000
750
500
250
0
0 20 40 60 80 100 120 140
Time (minutes)
Very High Positive Response
mRNA Cln1,2cyt
Cdk1Cln1,2nuc
Cdk1Cln1,2cyt
Cln1,2cyt
Cln3cyt
mRNA Cln1,2nuc
70
60
50
40
30
20
10
0
0 20 40 60 80 100 120 140
Time (minutes)
High Positive Response
mRNA Clb5,6cyt
Cdk1Clb5,6cyt
Cdk1Clb5,6-Sic1cyt
Sic1cyt
mRNA Clb5,6nuc
Clb5,6cyt
3.5
3.
2.5
2.
1.5
1.
0.5
0
0 20 40 60 80 100 120 140
Time (minutes)
Medium Positive Response
Cdk1Clb5,6nuc
SBF/MBFnuc
Cdk1Clb5,6-Sic1nuc
Cdk1Clb5,6-Sic1Pnuc
Cdk1nuc
Cdk1cyt
1.
0.8
0.6
0.4
0.2
0
0 20 40 60 80 100 120 140
Time (minutes)
Very High Negative Response
Far1cyt
SBF/MBF-Whi5nuc
Far1nuc
0
−5
−10
−15
−20
0 20 40 60 80 100 120 140
Time (minutes)
Medium Negative Response
Whi5cyt
Whi5nuc
0
−0.05
−0.1
−0.15
−0.2
−0.25
0 20 40 60 80 100 120 140
Time (minutes)
