# Supplementary material for: Cell Size at S Phase Initiation: An Emergent Property of the G1/S Network
Source: PLoS Comput Biol. 2007 Apr 13;3(4):e64. doi: 10.1371/journal.pcbi.0030064 (PMC1851985; doi:10.1371/journal.pcbi.0030064)

Figure S2A

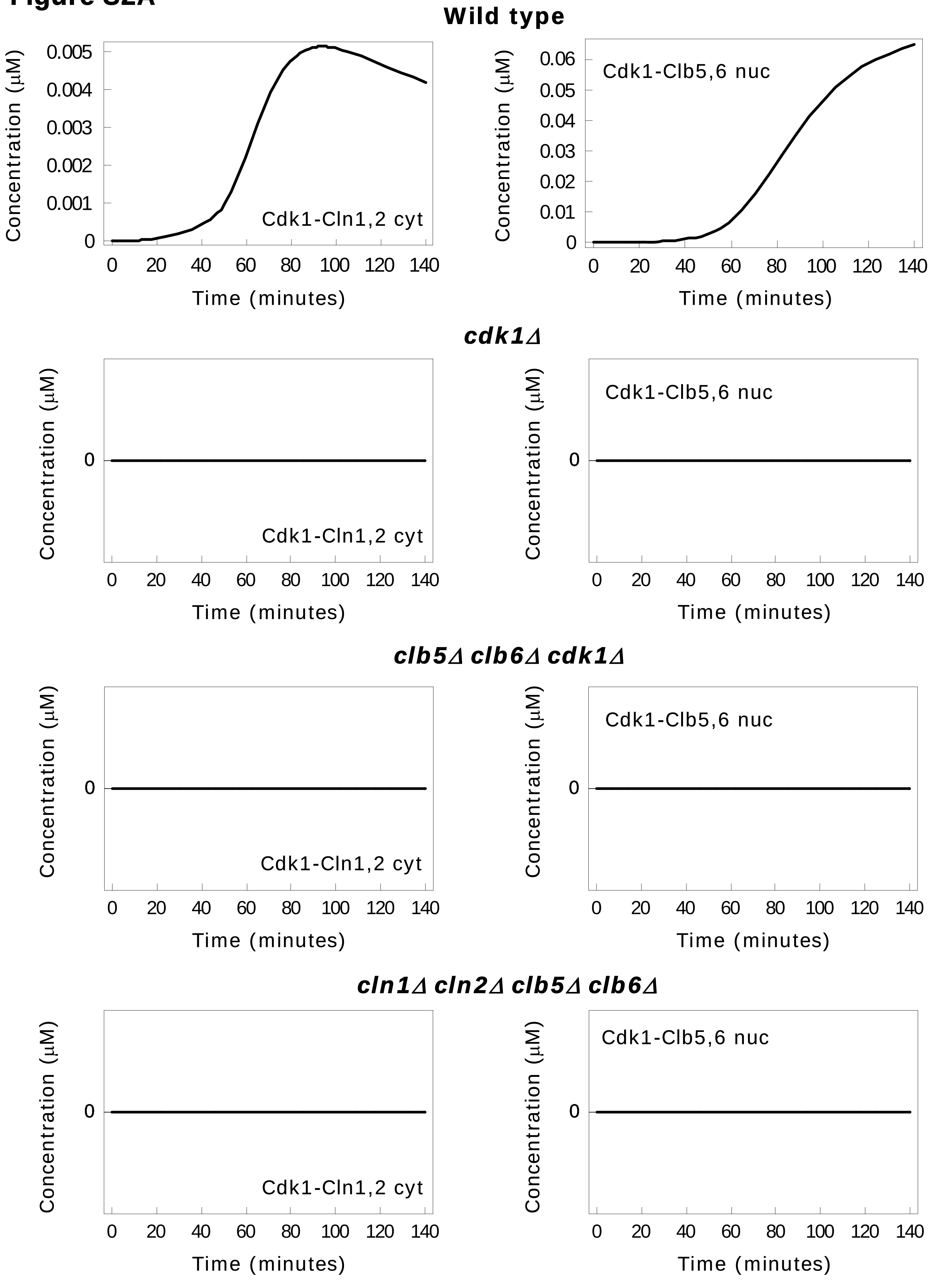
Wild type
Concentration (μM)
0.005
0.004
0.003
0.002
0.001
0
Cdk1-Cln1,2 cyt
0.06
0.05
0.04
0.03
0.02
0.01
Cdk1-Clb5,6 nuc
0 20 40 60 80 100 120 140
Time (minutes)
cdk1Δ
clb5Δ clb6Δ cdk1Δ
cln1Δ cln2Δ clb5Δ clb6Δ

Figure S2B

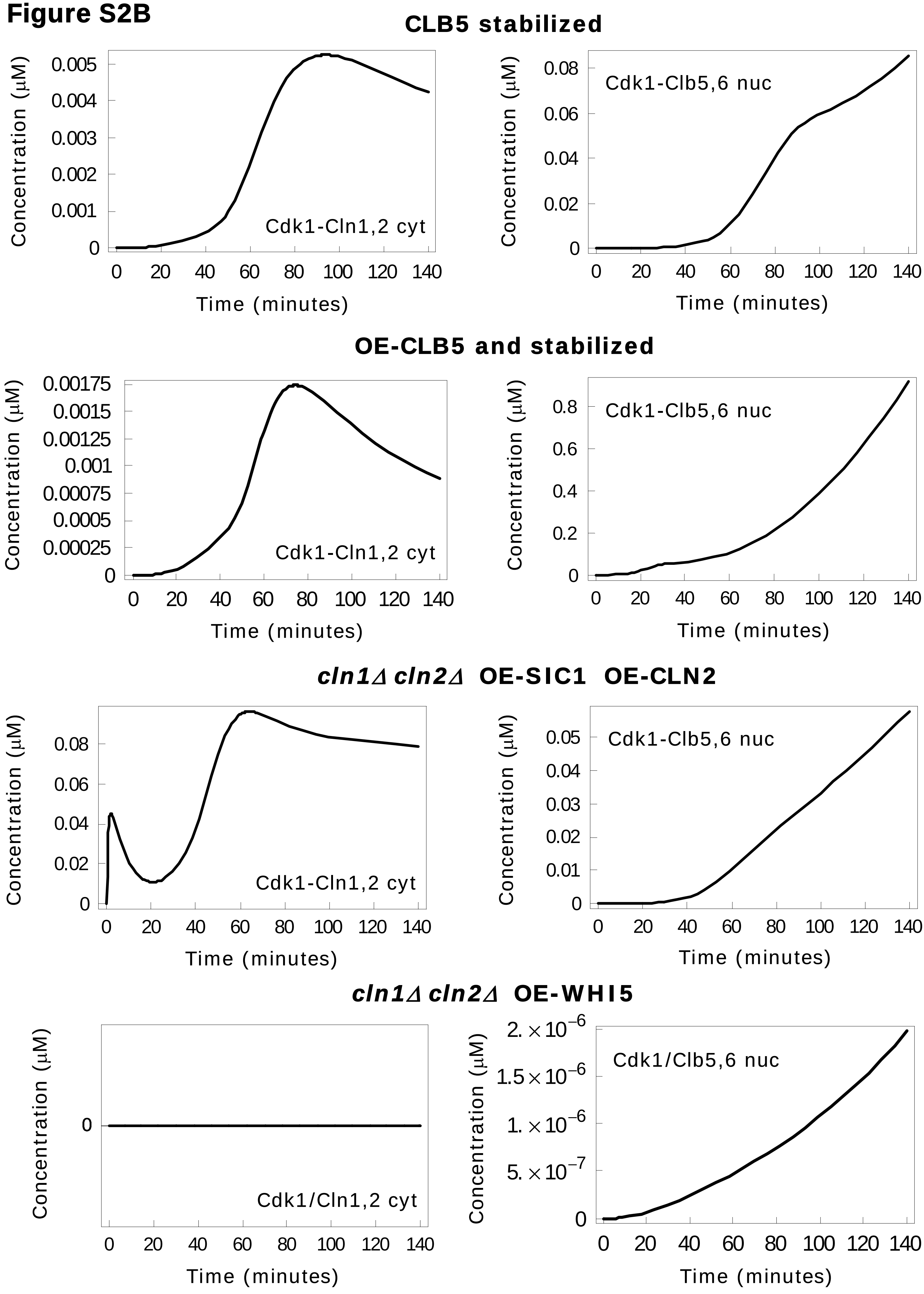
CLB5 stabilized
Cdk1-Cln1,2 cyt
Cdk1-Clb5,6 nuc
OE-CLB5 and stabilized
Cdk1-Cln1,2 cyt
Cdk1-Clb5,6 nuc
cln1Δ cln2Δ OE-SIC1 OE-CLN2
Cdk1-Cln1,2 cyt
Cdk1-Clb5,6 nuc
cln1Δ cln2Δ OE-WHI5
Cdk1/Cln1,2 cyt
Cdk1/Clb5,6 nuc
Concentration (μM)
Time (minutes)

Figure S2C

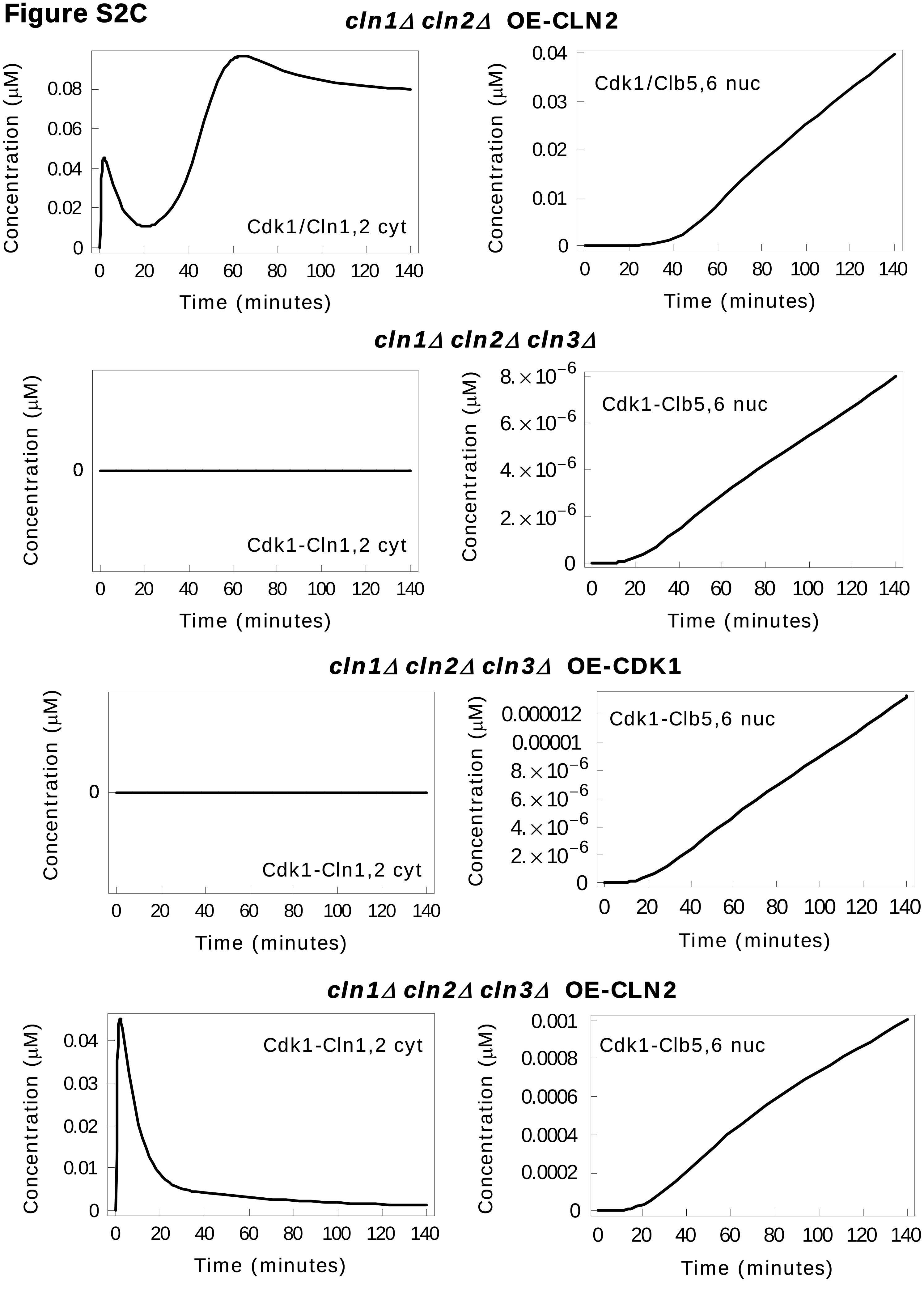

cln1Δ cln2Δ OE-CLN2
Cdk1/Cln1,2 cyt
Cdk1/Clb5,6 nuc
cln1Δ cln2Δ cln3Δ
Cdk1-Cln1,2 cyt
Cdk1-Clb5,6 nuc
cln1Δ cln2Δ cln3Δ OE-CDK1
Cdk1-Cln1,2 cyt
Cdk1-Clb5,6 nuc
cln1Δ cln2Δ cln3Δ OE-CLN2
Cdk1-Cln1,2 cyt
Cdk1-Clb5,6 nuc
Concentration (μM)
Time (minutes)

Figure S2D

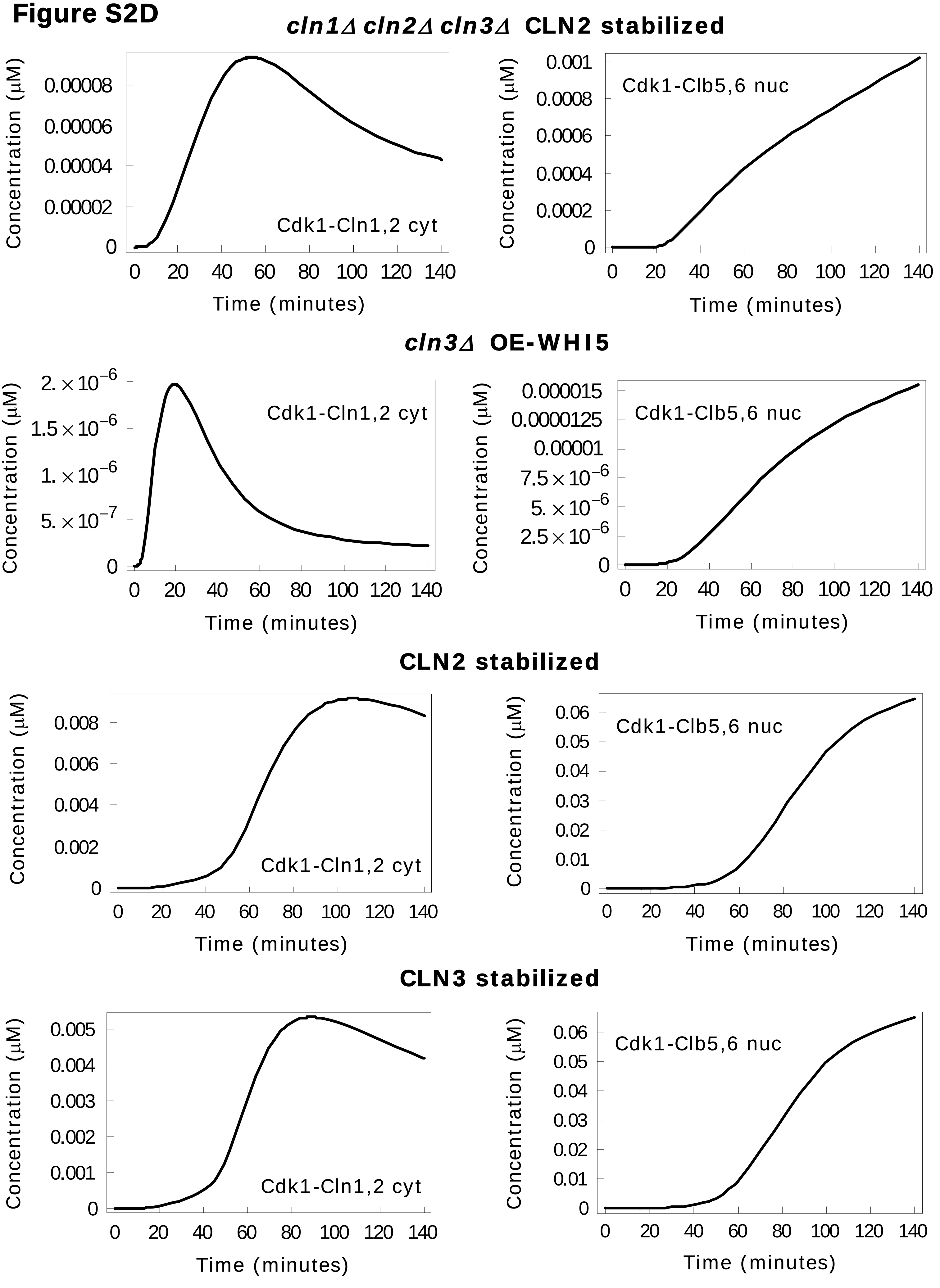
cln1Δ cln2Δ cln3Δ CLN2 stabilized
Cdk1-Cln1,2 cyt
Cdk1-Clb5,6 nuc
cln3Δ OE-WHI5
Cdk1-Cln1,2 cyt
Cdk1-Clb5,6 nuc
CLN2 stabilized
Cdk1-Cln1,2 cyt
Cdk1-Clb5,6 nuc
CLN3 stabilized
Cdk1-Cln1,2 cyt
Cdk1-Clb5,6 nuc
Concentration (μM)
Time (minutes)

Figure S2E

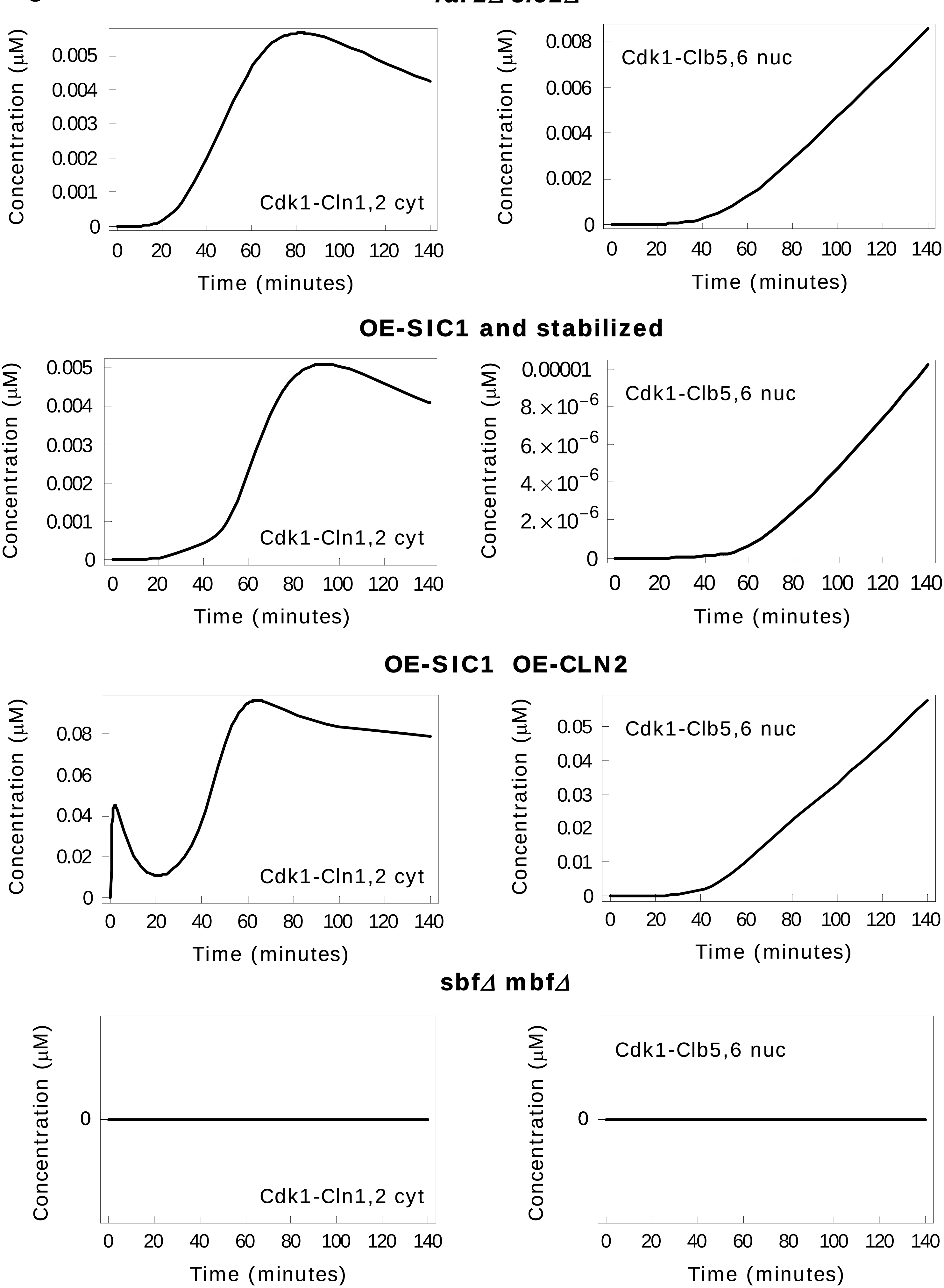
far1Δ sic1Δ
Cdk1-Cln1,2 cyt
Cdk1-Clb5,6 nuc
OE-SIC1 and stabilized
Cdk1-Cln1,2 cyt
Cdk1-Clb5,6 nuc
OE-SIC1 OE-CLN2
Cdk1-Cln1,2 cyt
Cdk1-Clb5,6 nuc
sbfΔ mbfΔ
Cdk1-Cln1,2 cyt
Cdk1-Clb5,6 nuc
Concentration (μM)
Time (minutes)

Figure S2F

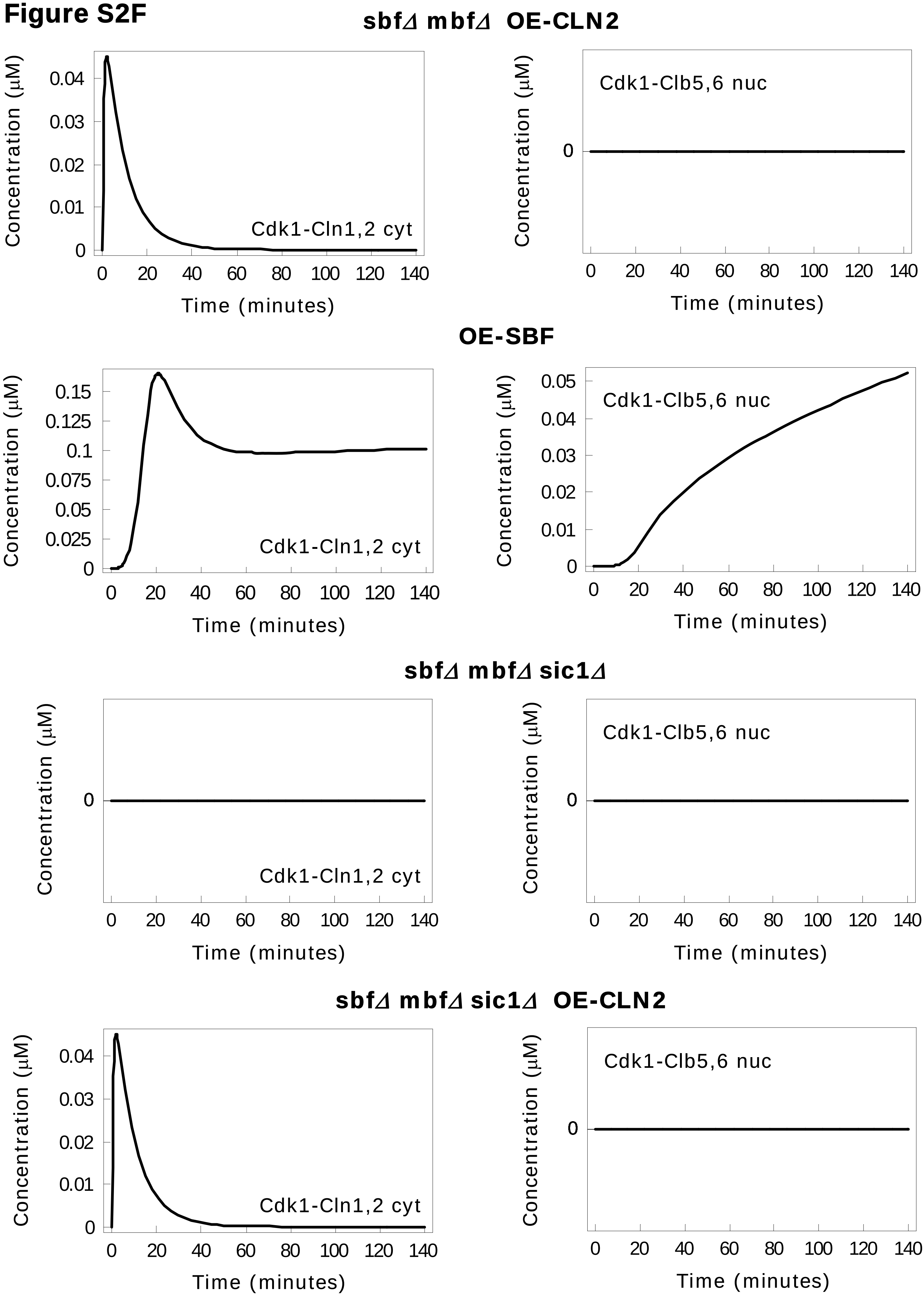
sbfΔ mbfΔ OE-CLN2
Cdk1-Cln1,2 cyt
Cdk1-Clb5,6 nuc
OE-SBF
Cdk1-Cln1,2 cyt
Cdk1-Clb5,6 nuc
sbfΔ mbfΔ sic1Δ
Cdk1-Cln1,2 cyt
Cdk1-Clb5,6 nuc
sbfΔ mbfΔ sic1Δ OE-CLN2
Cdk1-Cln1,2 cyt
Cdk1-Clb5,6 nuc
Concentration (μM)
Time (minutes)
0
20
40
60
80
100
120
140
0.01
0.02
0.03
0.04
0.025
0.05
0.075
0.1
0.125
0.15

Figure S2G

OE-MBF

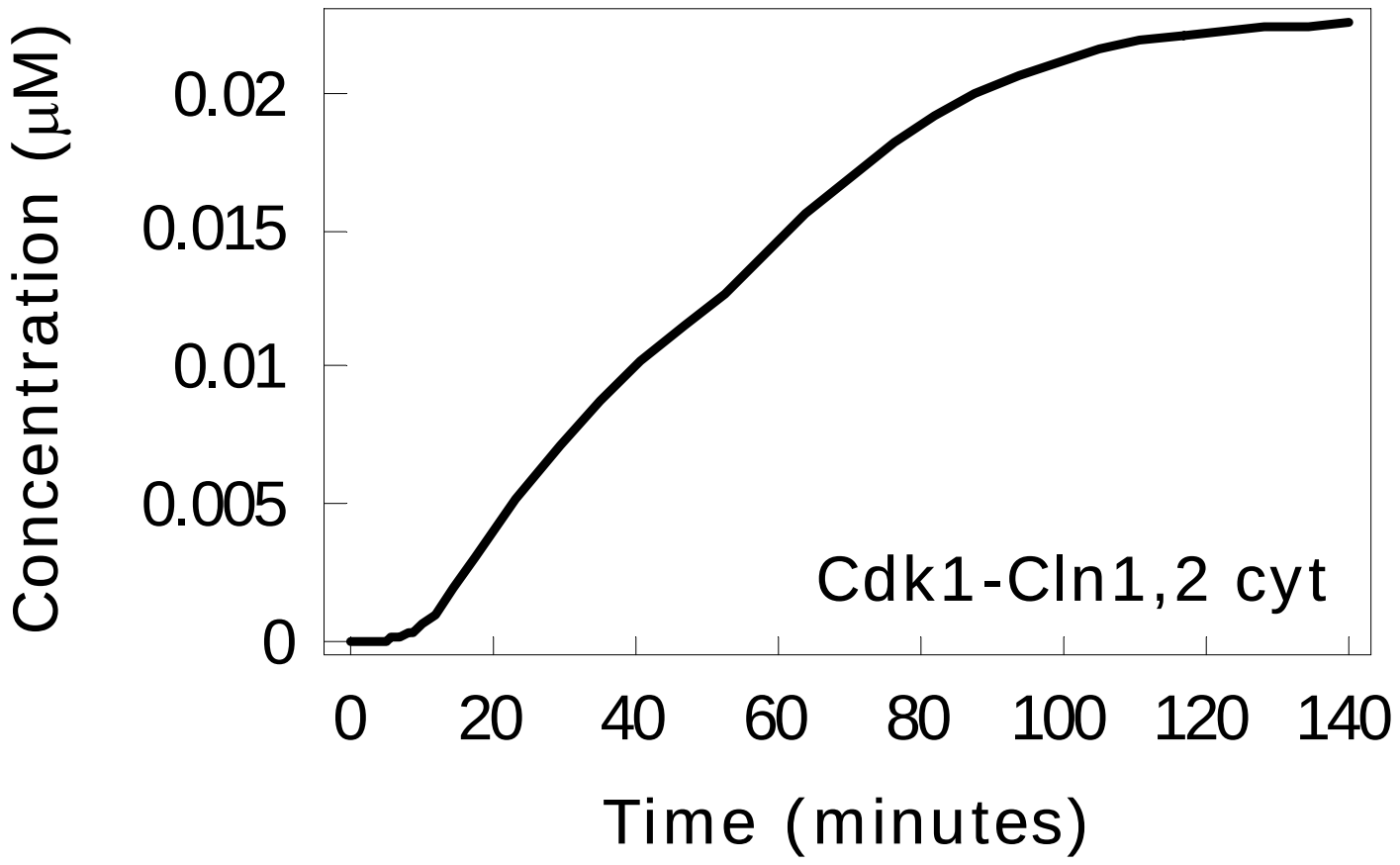

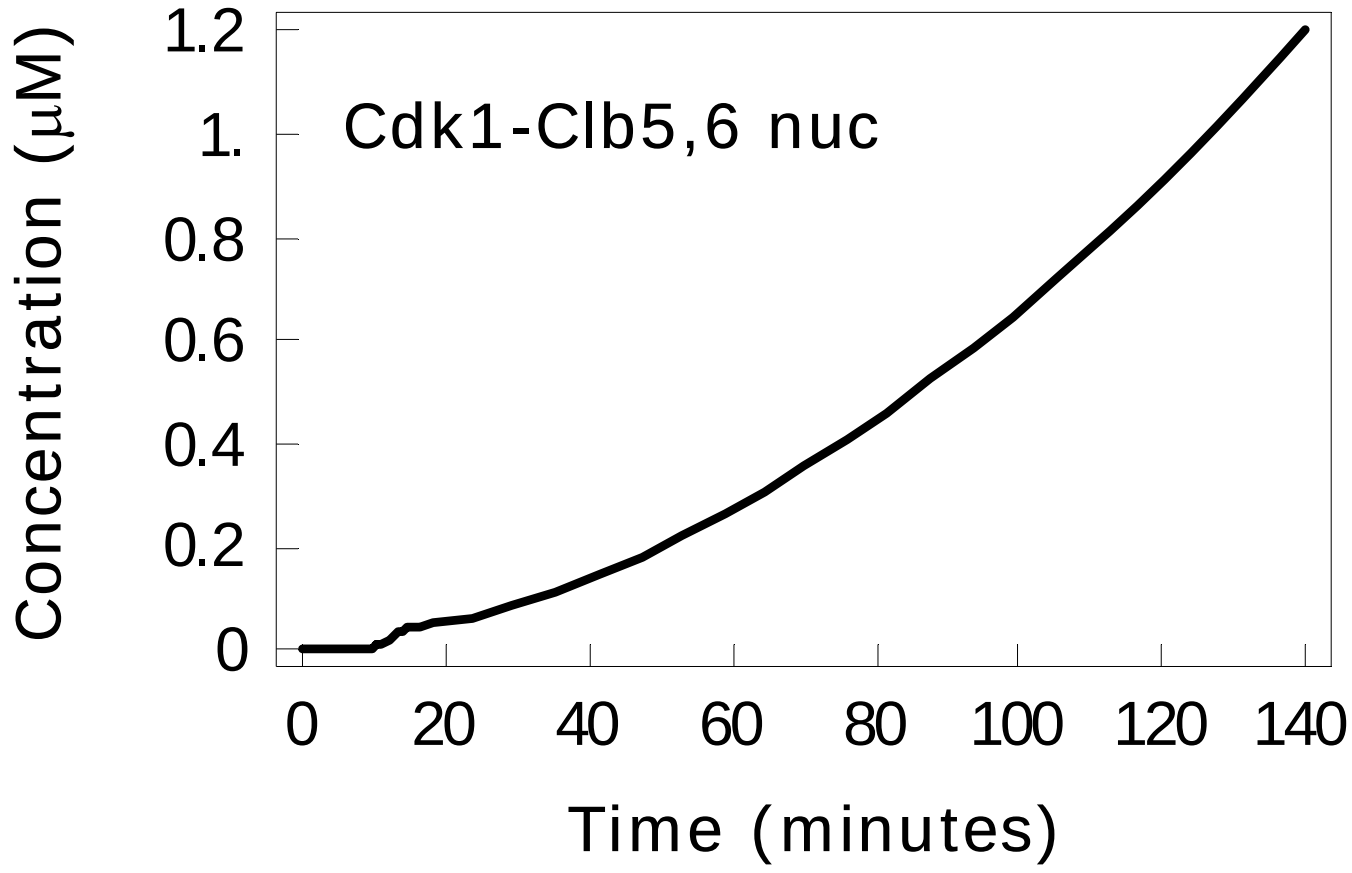

Supplement: Supplementary file 2 [file pcbi.0030064.sg002.pdf]
