# Supplementary material for: Cell Size at S Phase Initiation: An Emergent Property of the G1/S Network
Source: PLoS Comput Biol. 2007 Apr 13;3(4):e64. doi: 10.1371/journal.pcbi.0030064 (PMC1851985; doi:10.1371/journal.pcbi.0030064)

A

GLUCOSE – OE-FAR1

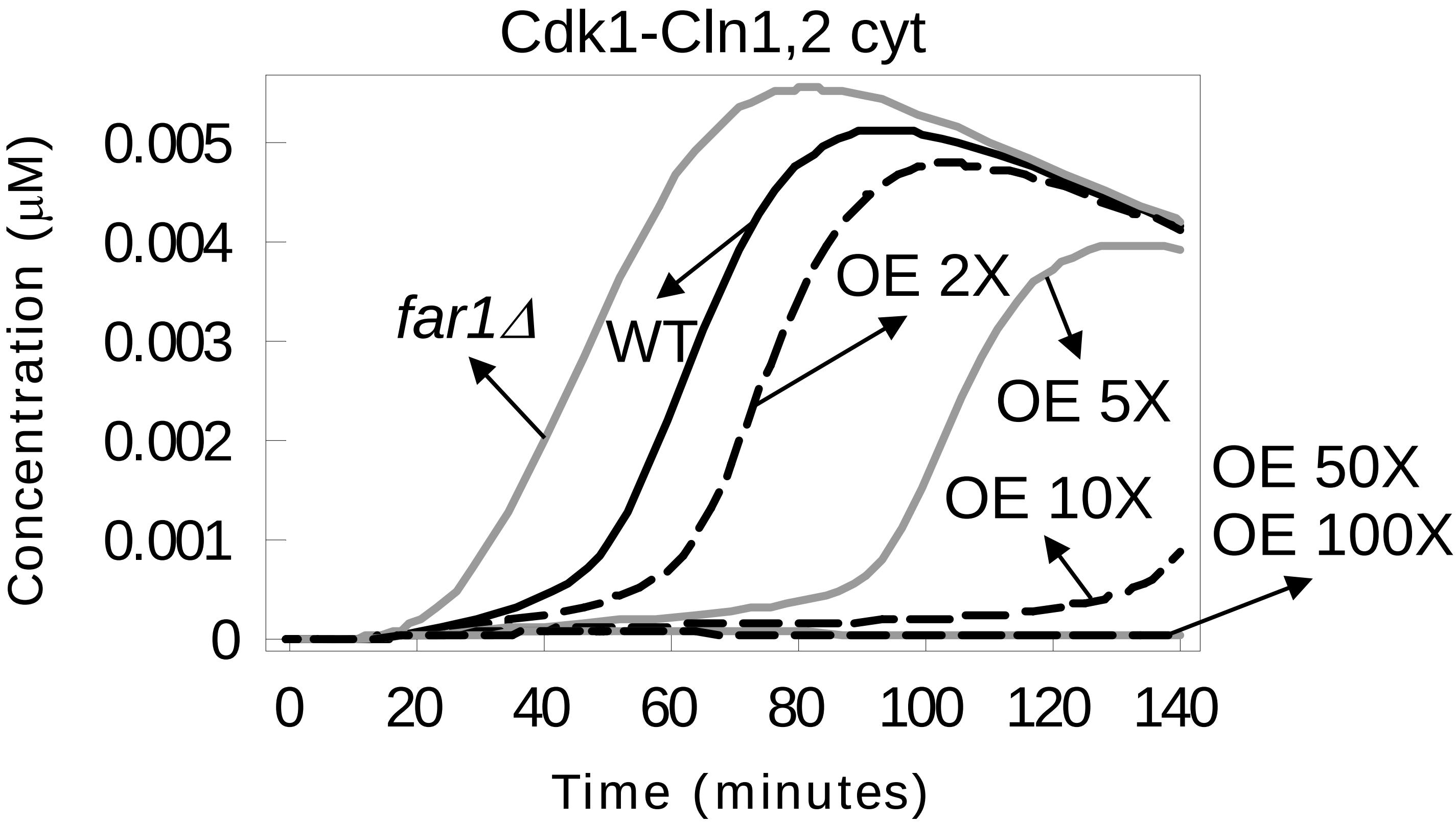

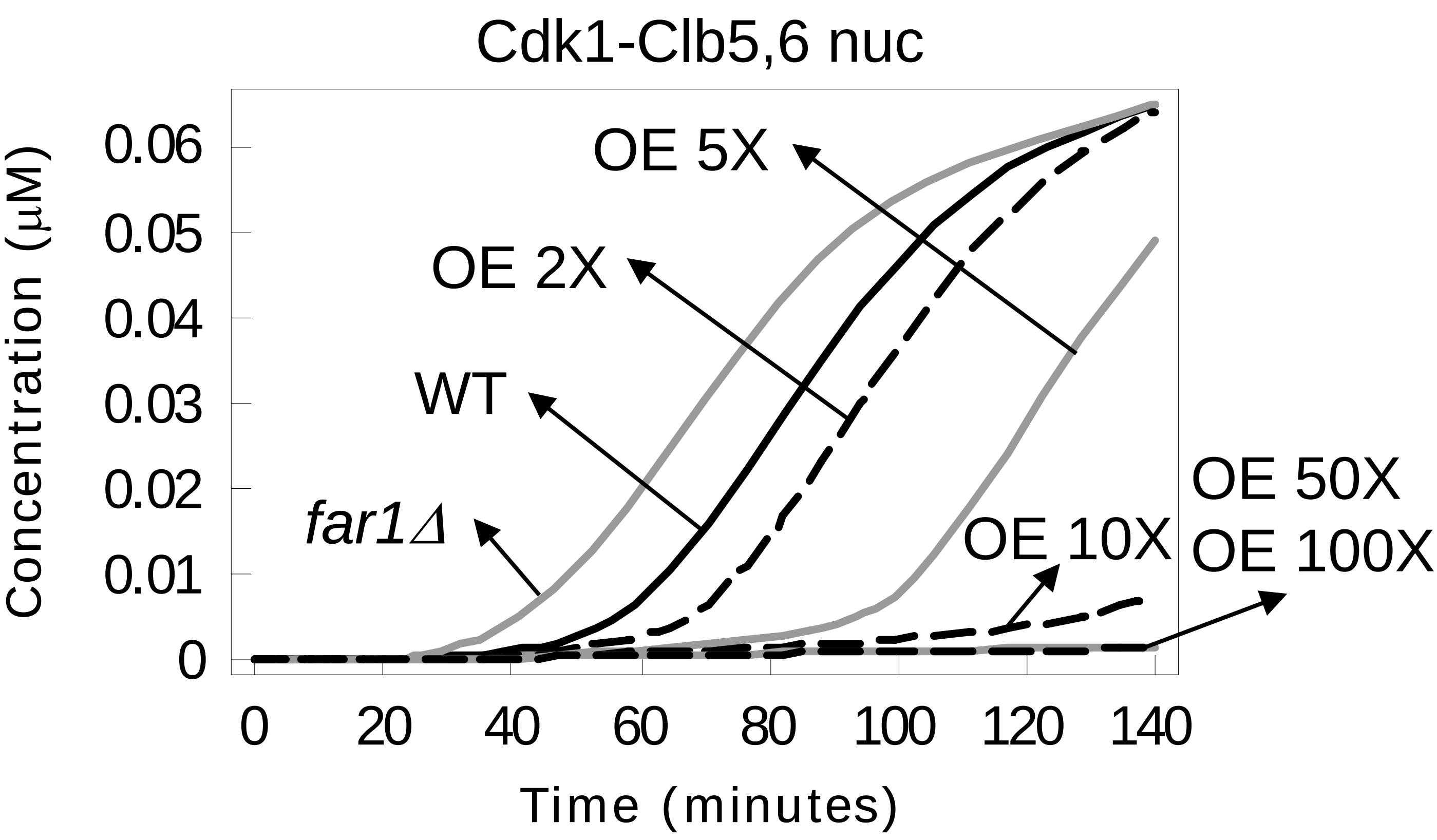

B

ETHANOL – OE-FAR1

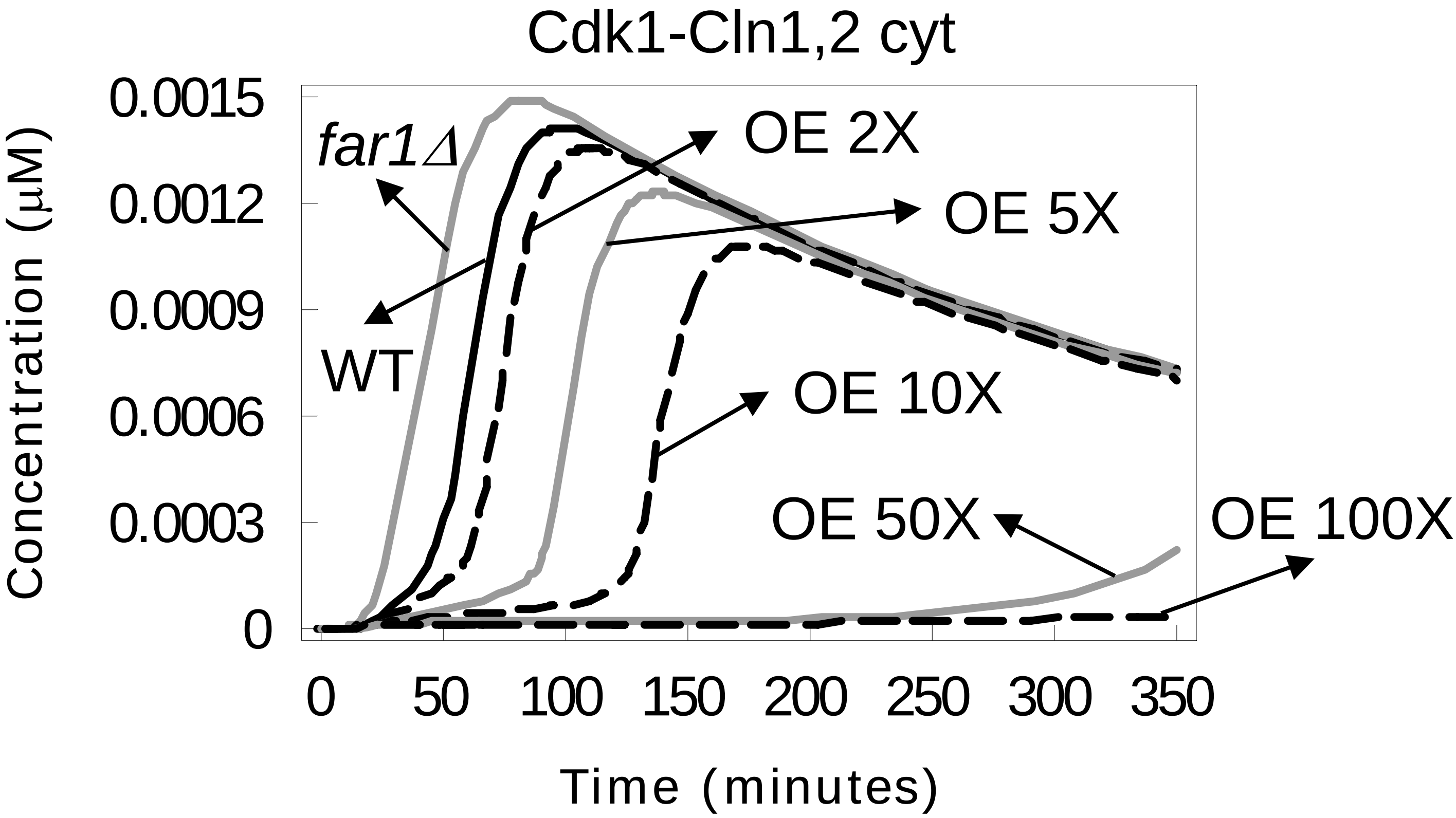

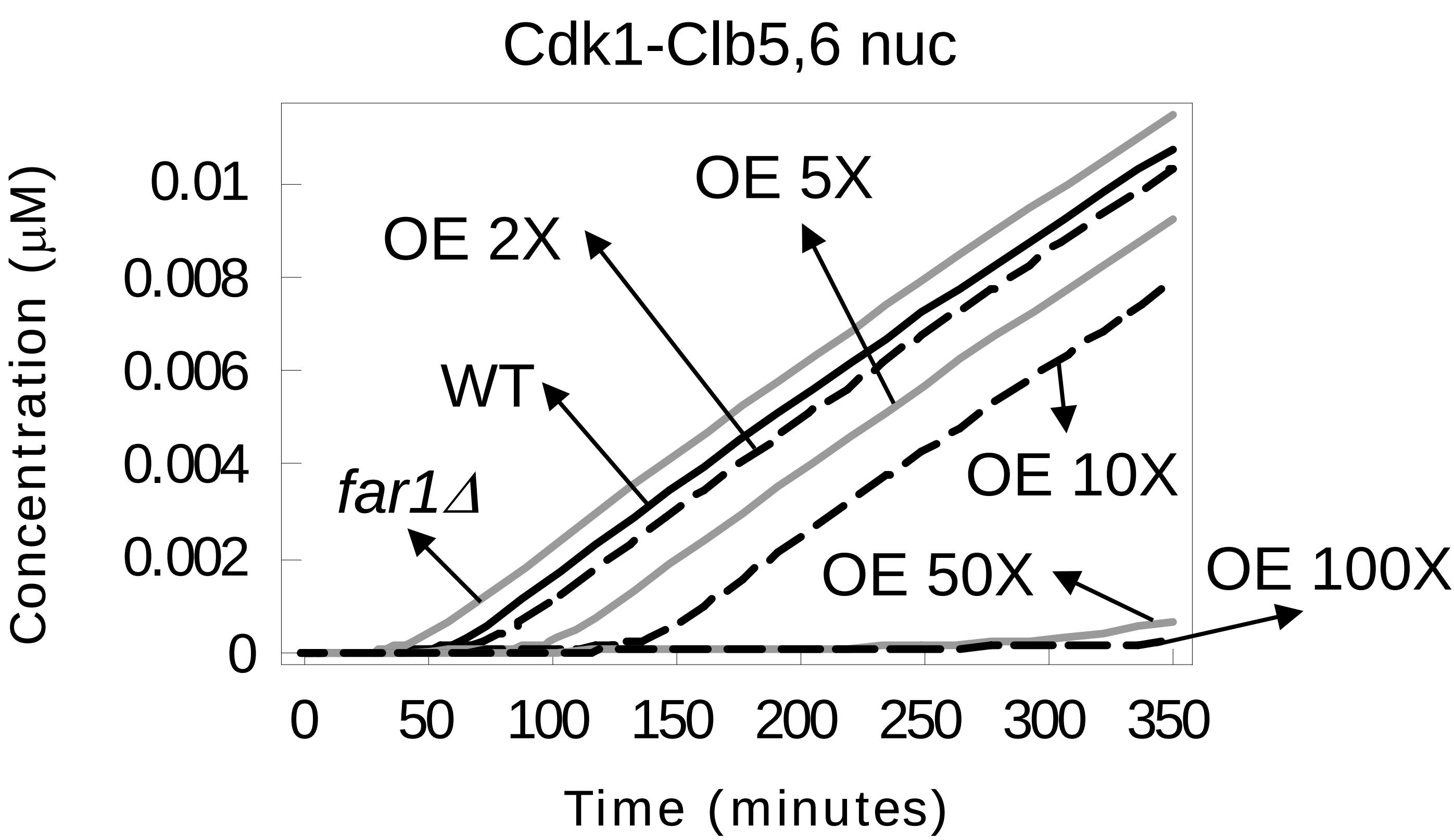

Supplement: Supplementary file 3 [file pcbi.0030064.sg003.pdf]
