# Supplementary material for: Cell Size at S Phase Initiation: An Emergent Property of the G1/S Network
Source: PLoS Comput Biol. 2007 Apr 13;3(4):e64. doi: 10.1371/journal.pcbi.0030064 (PMC1851985; doi:10.1371/journal.pcbi.0030064)
Supplement: Supplementary file 4 [file pcbi.0030064.st001.pdf]

Table S1

Response[1]		
**Rate constant**	**Budding**	**DNA replication**
k_1	Medium positive	Medium positive
k_2	–	High positive
k_3	Low positive	–
k_4	–	Medium positive
k_5	High negative	Very High negative
k_6	Very High positive	Very High positive
k_7	Medium positive	High positive
k_8	Medium negative	High negative
k_9	Medium negative	Very High positive
k_{10}	Low negative	–
k_{11}	–	Medium negative
k_{12}	Low negative	–
k_{13}	–	Medium negative
k_{14}	Low positive	Medium positive
k_{15}	Low negative	Medium negative
k_{16}	Low negative	Medium negative
k_{17}	Low positive	Medium positive
k_{18}	Low positive	High negative
k_{19}	Low positive	Medium positive
k_{20}	Low negative	Medium negative
k_{21}	Low negative	Medium negative
k_{22}	–	Medium positive
k_{25}	Medium positive	–
k_{29}	–	Medium negative
k_{35}	–	High positive
k_{42}	Medium negative	High negative
k_{43}	Medium positive	High positive
k_{44}	Medium positive	High positive
k_{45}	Medium negative	Medium negative
k_{46}	Low negative	Medium positive
k_{48}	–	Medium positive
k_{49}	Low positive	Medium positive
k_{53}	Medium positive	–

Response[1]		
**Initial concentration**	**Budding**	**DNA replication**
$Cdk1Cln3_{nuc}$	Very High positive	Very High positive
$Cdk1Cln3\text{-}Far1P_{nuc}$	Very High positive	Very High positive
$Cdk1Cln3\text{-}Far1_{nuc}$	Very High positive	Very High positive
$Cln3_{nuc}$	Very High positive	Very High positive
$Cln3_{cyt}$	High positive	Very High positive
mRNA $Cln1,2_{nuc}$	High positive	Very High positive
mRNA $Cln1,2_{cyt}$	High positive	Very High positive
$Cln1,2_{cyt}$	High positive	Very High positive
$Cdk1Cln1,2_{nuc}$	High positive	Very High positive
$Cdk1Cln1,2_{cyt}$	High positive	Very High positive
mRNA $Clb5,6_{cyt}$	–	High positive
mRNA $Clb5,6_{nuc}$	–	High positive
$Clb5,6_{cyt}$	–	High positive
$Cdk1Clb5,6_{cyt}$	–	High positive
$Sic1_{cyt}$	–	High positive
$Cdk1Clb5,6\text{-}Sic1_{cyt}$	–	High positive
$Cdk1\text{-}Clb5,6_{nuc}$	–	Medium positive
$Cdk1Clb5,6\text{-}Sic1_{nuc}$	–	Medium positive
$Cdk1Clb5,6\text{-}Sic1P_{nuc}$	–	Medium positive
$Cdk1_{cyt}$	–	Medium positive
$Cdk1_{nuc}$	Low positive	Medium positive
SBF/MBF_{nuc}	Low positive	Medium positive
$SBF/MBF\text{-}Whi5_{nuc}$	High negative	Very High positive
$Far1_{cyt}$	High negative	Very High negative
$Far1_{nuc}$	High negative	Very High negative
$Whi5_{nuc}$	High negative	Medium negative
$Whi5_{cyt}$	–	Medium negative

[1] Symbol – means negligible effect.
