# Supplementary material for: Cell Size at S Phase Initiation: An Emergent Property of the G1/S Network
Source: PLoS Comput Biol. 2007 Apr 13;3(4):e64. doi: 10.1371/journal.pcbi.0030064 (PMC1851985; doi:10.1371/journal.pcbi.0030064)
Supplement: Supplementary file 5 [file pcbi.0030064.st002.pdf]

Table S2

Mutant	Simulation result	Predicted phenotype	Observed phenotype (Ref.)
cdk1Δ	No formation of Cdk1-Cln1,2$_{cyt}$ and Cdk1-Clb5,6$_{nuc}$	Lethal. No budding and DNA replication	Lethal. The cells arrest as unbudded cells (1)
clb5Δ clb6Δ cdk1Δ	No formation of Cdk1-Cln1,2 $_{cyt}$ and Cdk1-Clb5,6$_{nuc}$	Lethal. No budding and DNA replication	Lethal (2)
cln1Δ cln2Δ clb5Δ clb6Δ	No formation of Cdk1-Cln1,2$_{cyt}$ and Cdk1-Clb5,6$_{nuc}$	Lethal. No budding and DNA replication	Lethal. The cells arrest in G_1 phase (3)
CLB5 stabilized	Wild type level of Cdk1-Cln1,2$_{cyt}$, and slight increase of the Cdk1-Clb5,6$_{nuc}$ level	Viable. Budding like wild type, and slight anticipation of DNA replication	Viable. The strain rescues the replication defect of the *clb5Δ* strain (4)
OE-CLB5 and stabilized	Decrease of the Cdk1-Cln1,2$_{cyt}$ level, and very high level (about 10X) of Cdk1-Clb5,6$_{nuc}$	Decrease of budding, and abnormal increase of DNA replication. Possibly lethal	Lethal (5)
cln1Δ cln2Δ OE-SIC1 OE-CLN2	Increase of the Cdk1-Cln1,2$_{cyt}$ level, and similar timing of Cdk1-Clb5,6$_{nuc}$ formation	Viable. Increase of budding, and DNA replication comparable to wild type	Viable. The strain suppresses the defects of the *cln1Δ cln2Δ* OE-SIC1 strain (6)
cln1Δ cln2Δ OE-WHI5	No formation of Cdk1-Cln1,2$_{cyt}$, and very strong decrease of the Cdk1-Clb5,6$_{nuc}$ level (four orders of magnitude)	Lethal. No budding and DNA replication	Lethal. Severely delayed growth (7)
cln1Δ cln2Δ OE-CLN2	Increased of the Cdk1-Cln1,2$_{cyt}$ level (about 15X), similar timing, but overall reduction (about 1/3) of Cdk1-Clb5,6$_{nuc}$ formation	Viable. Slight anticipation of budding, DNA replication should be ca. normal	Viable. The cells go through START immediately after cytokinesis (8)
cln1Δ cln2Δ cln3Δ	No formation of Cdk1-Cln1,2$_{cyt}$, and very strong decrease of the Cdk1-Clb5,6$_{nuc}$ level (three/four orders of magnitude)	Lethal. No budding and DNA replication	Lethal. The cells arrest the growth in G_1 phase (9)
cln1Δ cln2Δ cln3Δ OE-CDK1	No formation of Cdk1-Cln1,2$_{cyt}$, and very strong decrease of the Cdk1-Clb5,6$_{nuc}$ level (three orders of magnitude)	No budding and very strong delay in DNA replication. Lethal	Lethal. The phenotype of the triple cyclins mutant is not recovered (10)

cln1Δ cln2Δ cln3Δ OE-CLN2	Early formation of Cdk1-Cln1,2_{cyt}, and decrease of the Cdk1-Clb5,6_{nuc} level (one order of magnitude)	Viable. Anticipation of budding, and delay in DNA replication	Viable [11]
cln1Δ cln2Δ cln3Δ CLN2 stabilized	Strong decrease of the Cdk1-Cln1,2_{cyt} level, and decrease of the Cdk1-Clb5,6_{nuc} level (one order of magnitude)	Viable. Decrease of budding, and delay in DNA replication	Viable. The phenotype of the triple cyclins mutant is recovered [12]
cln3Δ OE-WHI5	Very strong decrease of the Cdk1-Cln1,2_{cyt} level, and very strong decrease of the Cdk1-Clb5,6_{nuc} level (three orders of magnitude)	Lethal. No budding, and very strong delay in DNA replication	Lethal. The cells arrest as unbudded cells [13]
CLN2 stabilized	Slight increase of the Cdk1-Cln1,2_{cyt} level, and similar timing of Cdk1-Clb5,6_{nuc} formation	Viable. Increased budding, and DNA replication comparable to wild type	Viable [14]
CLN3 stabilized<br><br>NOTE. *Lack of agreement on cell size likely originates from the fact that stable Cln3 mutants are more stable at the end of the cycle. The net result may thus be a larger Cln3 level in new-born cells, synthesis then proceeding as in wild type*	Level and timing of Cdk1-Cln1,2_{cyt} and Cdk1-Clb5,6_{nuc} formation like wild type	Viable. Budding and DNA replication comparable to wild type	Viable. No arrest of the cell cycle in G_1 phase, and small cell size [15]
far1Δ sic1Δ	Slight anticipation of the Cdk1-Cln1,2_{cyt} level, and decrease of the Cdk1-Clb5,6_{nuc} level (one order of magnitude)	Viable. Slight anticipation of budding, and delay in DNA replication	Viable [16]
OE-SIC1 and stabilized	Wild type level of Cdk1-Cln1,2_{cyt}, and very strong decrease of the Cdk1-Clb5,6_{nuc} level (three orders of magnitude)	Lethal. Budding like wild type, and very strong delay in DNA replication	Lethal. The cells arrest in G_1 phase [17]
OE-SIC1 OE-CLN2	Increase of the Cdk1-Cln1,2_{cyt} level, and similar timing of Cdk1-Clb5,6_{nuc} formation	Viable. Increase of budding, and slight delay in DNA replication	Viable. OE-CLN2 suppresses the toxicity of the OE-SIC1 strain [18]
sbfΔ mbfΔ	No formation of Cdk1-Cln1,2_{cyt} and Cdk1-Clb5,6_{nuc}	Lethal. No budding and DNA replication	Lethal [19]
sbfΔ mbfΔ OE-CLN2	Early formation of Cdk1-Cln1,2_{cyt}, and no	Viable. Anticipation of budding, DNA	Viable. The strain rescues the *sbfΔ*

NOTE. *To get agreement with experimental data, we must assume that synthesis of Clb3,4 (not included in the model) must start at some point*	Cdk1-Clb5,6_{nuc} formation	replication delayed because synthesis of Clb3,4 (not included in the model) would be required to get (delayed) S phase	lethal phenotype [20]
OE-SBF<br><br>NOTE. *SBF and MBF are not differentiated in our model, and we consider OE-SBF increasing the rate of CLN1,2 transcription*	Strong increase of Cdk1-Cln1,2_{cyt} level, and slight anticipation of Cdk1-Clb5,6_{nuc} formation	Viable. Strong anticipation of budding, and slight anticipation in DNA replication	Viable. The overexpression results in small cell size [21]
sbfΔ mbfΔ sic1Δ	No formation of Cdk1-Cln1,2_{cyt} and Cdk1-Clb5,6_{nuc}	Lethal. No budding and DNA replication	Lethal [22]
sbfΔ mbfΔ sic1Δ OE-CLN2	Early formation of Cdk1-Cln1,2_{cyt}, and no Cdk1-Clb5,6_{nuc} formation	Viable. Anticipation of budding, DNA replication delayed because synthesis of Clb3,4 (not included in the model) would be required to get (delayed) S phase	Viable [23]
OE-MBF<br><br>NOTE. *SBF and MBF are not differentiated in our model, and we consider OE-MBF increasing the rate of CLB5,6 transcription*	Early formation of Cdk1-Cln1,2_{cyt}, and very high level of Cdk1-Clb5,6_{nuc}	Lethal. Anticipation of budding, and abnormal increase of DNA replication	Lethal [24]

References

1

a) Giaever G, Chu AM, Ni L, Connelly C, Riles L, et al. (2002) Functional profiling of the *Saccharomyces cerevisiae* genome. Nature 418: 387–391.

2

a) Segal M, Clarke DJ, Reed SI (1998) Clb5-associated kinase activity is required early in the spindle pathway for correct preanaphase nuclear positioning in *Saccharomyces cerevisiae*. J Cell Biol 143: 135–145.
b) Cross FR, Jacobson MD (2000) Conservation and function of a potential substrate-binding domain in the yeast Clb5 B-type cyclin. Mol Cell Biol 20: 4782–4790.

3

a) Dirick L, Bohm T, Nasmyth K (1995) Roles and regulation of Cln-Cdc28 kinases at the start of the cell cycle of *Saccharomyces cerevisiae*. EMBO J 14: 4803–4813.

4

a) Cross FR, YusteRojas M, Gray S, Jacobson MD (1999) Specialization and targeting of B-type cyclins. Mol Cell 4: 11–19.

5

a) Cross FR, YusteRojas M, Gray S, Jacobson MD (1999) Specialization and targeting of B-type cyclins. Mol Cell 4: 11–19.
b) Jacobson MD, Gray S, Yuste-Rojas M, Cross FR (2000) Testing cyclin specificity in the exit from mitosis. Mol Cell Biol 20: 4483–4493.

6

a) Cross FR, Archambault V, Miller M, Klovstad M (2002) Testing a mathematical model of the yeast cell cycle. Mol Biol Cell 13: 52–70.

7

a) Costanzo M, Nishikawa JL, Tang X, Millman JS, Schub O, Breitkreuz K, Dewar D, Rupes I, Andrews B, Tyers M (2004) CDK activity antagonizes Whi5, an inhibitor of G1/S transcription in yeast. Cell 117: 899–913.

8

a) Cross FR, Archambault V, Miller M, Klovstad M (2002) Testing a mathematical model of the yeast cell cycle. Mol Biol Cell 13: 52–70.

9

a) Richardson HE, Wittenberg C, Cross F, Reed SI (1989) An essential G1 function for cyclin-like proteins in yeast. Cell 59: 1127–1133.
b) Schneider BL, Yang QH, Futcher AB (1996) Linkage of replication to start by the Cdk inhibitor Sic1. Science 272: 560–562.
c) Tyers M (1996) The cyclin-dependent kinase inhibitor p40SIC1 imposes the requirement for Cln G1 cyclin function at Start. Proc Natl Acad Sci USA 93: 7772–7776.

10

a) Leopold P, O'Farrell PH (1991) An evolutionarily conserved cyclin homolog from *Drosophila* rescues yeast deficient in G1 cyclins. Cell 66: 1207–1216.

11

a) Valdivieso MH, Sugimoto K, Jahng KY, Fernandes PM, Wittenberg C (1993) FAR1 is required for posttranscriptional regulation of CLN2 gene expression in response to mating pheromone. Mol Cell Biol 13: 1013–1022.

12

a) Lanker S, Valdivieso MH, Wittenberg C (1996) Rapid degradation of the G1 cyclin Cln2 induced by CDK-dependent phosphorylation. Science 271: 1597–1601.

13

a) Costanzo M, Nishikawa JL, Tang X, Millman JS, Schub O, Breitkreuz K, Dewar D, Rupes I, Andrews B, Tyers M (2004) CDK activity antagonizes Whi5, an inhibitor of G1/S transcription in yeast. Cell 117: 899–913.

14

a) YPD (Yeast Proteome Database) and SGD (*Saccharomyces* Genome Database): information without citation.

15

a) YPD (Yeast Proteome Database) and SGD (*Saccharomyces* Genome Database): information without citation.

16

a) Alberghina L, Rossi RL, Querin L, Wanke V, Vanoni M (2004) A cell sizer network involving Cln3 and Far1 controls entrance into S phase in the mitotic cycle of budding yeast. J Cell Biol 167: 433–443.

17

a) Verma R, Annan RS, Huddleston MJ, Carr SA, Reynard G, Deshaies RJ (1997) Phosphorylation of Sic1p by G1 Cdk required for its degradation and entry into S phase. Science 278: 455–460.

18

a) Huang KN, Odinsky SA, Cross FR (1997) Structure-function analysis of the *Saccharomyces cerevisiae* G1 cyclin Cln2. Mol Cell Biol 17: 4654–4666.

19

a) Breeden L, Nasmyth K (1987) Cell cycle control of the yeast HO gene: cis- and trans-acting regulators. Cell 48: 389–397.
b) Nasmyth K, Dirick L (1991) The role of SW14 and SW16 in the activity of G1 cyclins in yeast. Cell 66: 995–1013.
c) Ogas J, Andrews BJ, Herskowitz I (1991) Transcriptional activation of CLN1, CLN2, and a putative new G1 cyclin (HCS26) by SWI4, a positive regulator of G1-specific transcription. Cell 66: 1015–1026.
d) Edgington NP, Futcher B (2001) Relationship between the function and the location of G1 cyclins in *S. cerevisiae*. J Cell Sci 114: 4599–4611.

e) Betz JL, Chang M, Washburn TM, Porter SE, Mueller CL, Jaehning JA (2002) Phenotypic analysis of Paf1/RNA polymerase II complex mutations reveals connections to cell cycle regulation, protein synthesis, and lipid and nucleic acid metabolism. Mol Genet Genomics 268: 272–285.

20

a) Nasmyth K, Dirick L (1991) The role of SW14 and SW16 in the activity of G1 cyclins in yeast. Cell 66: 995–1013.
b) Ogas J, Andrews BJ, Herskowitz I (1991) Transcriptional activation of CLN1, CLN2, and a putative new G1 cyclin (HCS26) by SWI4, a positive regulator of G1-specific transcription. Cell 66: 1015–1026.
c) Moll T, Dirick L, Auer H, Bonkovsky J, Nasmyth K (1992) SWI6 is a regulatory subunit of two different cell cycle START-dependent transcription factors in *Saccharomyces cerevisiae*. J Cell Sci Suppl 16: 87–96.
d) Koch C, Moll T, Neuberg M, Ahorn H, Nasmyth K (1993) A role for the transcription factors Mbp1 and Swi4 in progression from G1 to S phase. Science 261: 1551–1557.
e) Edgington NP, Futcher B (2001) Relationship between the function and the location of G1 cyclins in *S. cerevisiae*. J Cell Sci 114: 4599–4611.

21

a) Breeden L, Mikesell G (1994) Three independent forms of regulation affect expression of HO, CLN1 and CLN2 during the cell cycle of *Saccharomyces cerevisia*e. Genetics 138: 1015–1024.
b) Dirick L, Bohm T, Nasmyth K (1995) Roles and regulation of Cln-Cdc28 kinases at the start of the cell cycle of *Saccharomyces cerevisiae*. EMBO J 14: 4803–4813.
c) Toone WM, Aerne BL, Morgan BA, Johnston LH (1997) Getting started: regulating the initiation of DNA replication in yeast. Annu Rev Microbiol 51: 125–149.

22

a) Wijnen H, Futcher B (1999) Genetic analysis of the shared role of CLN3 and BCK2 at the G(1)-S transition in *Saccharomyces cerevisiae*. Genetics 153: 1131–1143.
b) Edgington NP, Futcher B (2001) Relationship between the function and the location of G1 cyclins in S. cerevisiae. J Cell Sci 114: 4599–4611.

23

a) Wijnen H, Futcher B (1999) Genetic analysis of the shared role of CLN3 and BCK2 at the G(1)-S transition in *Saccharomyces cerevisiae*. Genetics 153: 1131–1143.

24

a) Bouquin N, Johnson AL, Morgan BA, Johnston LH (1999) Association of the cell cycle transcription factor Mbp1 with the Skn7 response regulator in budding yeast. Mol Biol Cell 10: 3389–3400.
