# Supplementary material for: Cell Size at S Phase Initiation: An Emergent Property of the G1/S Network
Source: PLoS Comput Biol. 2007 Apr 13;3(4):e64. doi: 10.1371/journal.pcbi.0030064 (PMC1851985; doi:10.1371/journal.pcbi.0030064)
Supplement: Supplementary file 6 [file pcbi.0030064.st003.pdf]

Table S3

Mutant	Rate constant	Initial concentration
OE-CLN3	k_6 = 100*0.00001 μM*min^{-1}	$cln3_{cyt}[0]$ = 100*0.000485 μM
far1Δ	k_5 = 0	$far1_{cyt}[0]$ = 0
OE-FAR1	k_5 = 100*0.000042 μM*min^{-1}	$far1_{cyt}[0]$ = 100*0.0037926 μM
whi5Δ	k_8 = 0	$whi5_{cyt}[0]$ = 0<br>$sbfwhi5_{nuc}[0]$ = 0<br>$sbf_{nuc}[0]$ = 0.025544 μM
OE-WHI5	k_8 = 100*0.00004 μM*min^{-1}	$whi5_{cyt}[0]$ = 100*0.073564 μM
sic1Δ	k_9 = 0	$sic1_{cyt}[0]$ = 0
OE-SIC1	k_9 = 100*0.00005 μM*min^{-1}	$sic1_{cyt}[0]$ = 100*0.039234 μM
cdk1Δ	k_7 = 0	$cdk1_{cyt}[0]$ = 0<br>$cdk1_{nuc}[0]$ = 0
clb5Δ clb6Δ cdk1Δ	k_2 = 0<br>k_7 = 0	$cdk1_{cyt}[0]$ = 0<br>$cdk1_{nuc}[0]$ = 0
cln1Δ cln2Δ clb5Δ clb6Δ	k_1 = 0<br>k_2 = 0	
CLB5 stabilized	k_{13} = 0	
OE-CLB5 and stabilized	k_2 = 2 min^{-1}<br>k_{13} = 0	$clb5,6_{cyt}[0]$ = 0.1 μM
cln1Δ cln2Δ OE-SIC1 OE-CLN2	k_1 = 2 min^{-1}<br>k_9 = 100*0.00005 μM*min^{-1}	$sic1_{cyt}[0]$ = 100*0.039234 μM<br>$cln1,2_{cyt}[0]$ = 0.1 μM
cln1Δ cln2Δ OE-WHI5	k_1 = 0<br>k_8 = 100*0.00004 μM*min^{-1}	$whi5_{cyt}[0]$ = 100*0.073564 μM
cln1Δ cln2Δ OE-CLN2	k_1 = 2 min^{-1}	$cln1,2_{cyt}[0]$ = 0.1 μM
cln1Δ cln2Δ cln3Δ	k_1 = 0<br>k_6 = 0	$cln3_{cyt}[0]$ = 0
cln1Δ cln2Δ cln3Δ OE-CDK1	k_1 = 0<br>k_6 = 0<br>k_7 = 100*0.01 μM*min^{-1}	$cln3_{cyt}[0]$ = 0<br>$cdk1_{cyt}[0]$ = 100*0.333333 μM
cln1Δ cln2Δ cln3Δ OE-CLN2	k_1 = 2 min^{-1}<br>k_6 = 0	$cln3_{cyt}[0]$ = 0<br>$cln1,2_{cyt}[0]$ = 0.1 μM
cln1Δ cln2Δ cln3Δ CLN2 stabilized	k_6 = 0<br>k_{12} = 0	$cln3_{cyt}[0]$ = 0
cln3Δ OE-WHI5	k_6 = 0<br>k_8 = 100*0.00004 μM*min^{-1}	$cln3_{cyt}[0]$ = 0<br>$whi5_{cyt}[0]$ = 100*0.073564 μM
CLN2 stabilized	k_{12} = 0	
CLN3 stabilized	k_{15} = 0<br>k_{20} = 0	

far1Δ sic1Δ	$k_5 = 0$<br>$k_9 = 0$	$far1_{cyt}[0] = 0$<br>$sic1_{cyt}[0] = 0$
OE-SIC1 and stabilized	$k_9 = 100*0.00005$ μM*min^{-1}<br>$k_{18} = 0$	$sic1_{cyt}[0] = 100*0.039234$ μM
OE-SIC1 OE-CLN2	$k_9 = 100*0.00005$ μM*min^{-1}<br>$k_1 = 2$ min^{-1}	$sic1_{cyt}[0] = 100*0.039234$ μM<br>$cln1,2_{cyt}[0] = 0.1$ μM
sbfΔ mbfΔ	$k_1 = 0$<br>$k_2 = 0$	$sbfwhi5_{nuc}[0] = 0$
sbfΔ mbfΔ<br>OE-CLN2	$k_2 = 0$<br>$k_1 = 2$ min^{-1}	$sbfwhi5_{nuc}[0] = 0$<br>$cln1,2_{cyt}[0] = 0.1$ μM
OE-SBF	$k_1 = 2$ min^{-1}	$sbfwhi5_{nuc}[0] = 100*0.025544$ μM
sbfΔ mbfΔ sic1Δ	$k_1 = 0$<br>$k_2 = 0$<br>$k_9 = 0$	$sbfwhi5_{nuc}[0] = 0$<br>$sic1_{cyt}[0] = 0$
sbfΔ mbfΔ sic1Δ<br>OE-CLN2	$k_2 = 0$<br>$k_9 = 0$<br>$k_1 = 2$ min^{-1}	$sbfwhi5_{nuc}[0] = 0$<br>$sic1_{cyt}[0] = 0$<br>$cln1,2_{cyt}[0] = 0.1$ μM
OE-MBF	$k_2 = 2$ min^{-1}	$sbfwhi5_{nuc}[0] = 100*0.025544$ μM

**External signals**	**Rate constant**	**Initial concentration**
Pheromones	$k_5 = 5*0.000042$ μM*min^{-1}<br>$k_{14} = 0$<br>$k_{19} = 0$	$far1_{cyt}[0] = 5*0.0037926$ μM
Hog1 cascade	$k_{18} = 0$<br>$k_1 = 0.003523$ min^{-1}	
