# Supplementary material for: Cell Size at S Phase Initiation: An Emergent Property of the G1/S Network
Source: PLoS Comput Biol. 2007 Apr 13;3(4):e64. doi: 10.1371/journal.pcbi.0030064 (PMC1851985; doi:10.1371/journal.pcbi.0030064)
Supplement: Supplementary file 7 [file pcbi.0030064.st004.pdf]

Table S4

Relevant genotype	Growth medium	Estimated P_S	Experimental [Ref]
Wild type	glucose	1.54	Standard
cln3Δ	glucose	–	Increase in cell size and P_S in comparison to wild type [1]
OE-CLN3	glucose	1.26	Decrease in cell size and P_S in comparison to wild type [2]
far1Δ	glucose	1.44	Minor, but reproducible decrease in P_S in comparison to wild type (detectable in synchronous cultures) [3]
OE-FAR1 2X	glucose	1.63	Minor, but reproducible increase in P_S in comparison to wild type [4]
whi5Δ	glucose	1.20	Decrease in cell size [5]
OE-WHI5	glucose	3.31	Increase in cell size [6]
sic1Δ	glucose	6.57	Larger cells, abnormal morphology [7]
OE-SIC1	glucose	1.50	Size and morphology similar to wild type [8]
Wild type	ethanol	1.20	Standard
OE-FAR1 10X	ethanol	1.42	Increase in cell size and P_S in comparison to wild type [4]

Relevant genotype	Wild type	*cln3Δ*
"normal" parameters	1.54	–
k_{35} = 0.01 min^{-1}	1.46	2.02
k_{48} = 0.1 min^{-1}	1.53	–
k_{35} = 0.01 min^{-1} and k_{48} = 0.1 min^{-1}	1.45	2.00

References

1

a) Dirick L, Bohm T, Nasmyth K (1995) Roles and regulation of Cln/Cdc28 kinases at the start of the cell cycle of *Saccharomyces cerevisiae*. EMBO J 14: 4803–4813.
b) Bryan BA, McGrew E, Lu Y, Polymenis M (2004) Evidence for control of nitrogen metabolism by a START-dependent mechanism in *Saccharomyces cerevisiae*. Mol Genet Genomics 271: 72–81.

2

a) Tyers M, Tokiwa G, Nash R, Futcher B (1992) The Cln3-Cdc28 kinase complex of *S. cerevisiae* is regulated by proteolysis and phosphorylation. EMBO J 11: 1773–1784.
b) Tyers M, Tokiwa G, Futcher B (1993) Comparison of the *Saccharomyces cerevisiae* G1 cyclins: Cln3 may be an upstream activator of Cln1, Cln2 and other cyclins. EMBO J 12: 1955–1968.
c) Futcher B (1996) Cyclins and the wiring of the yeast cell cycle. Yeast 12: 1635–1646.
d) Cross FR, Archambault V, Miller M, Klovstad M (2002) Testing a mathematical model of the yeast cell cycle. Mol Biol Cell 13: 52–70.

3

a) Wijnen, H., Landman, A., and Futcher, B. The G(1) Cyclin Cln3 Promotes Cell Cycle Entry via the Transcription Factor Swi6. Mol Cell Biol 22, 4402-4418. (2002).
b) Alberghina, L., Rossi, R.L., Querin, L., Wanke, V. & Vanoni, M. A cell sizer network involving Cln3 and Far1 controls entrance into S phase in the mitotic cycle of budding yeast. *J. Cell Biol.* **167**, 433–443 (2004).

4

a) Alberghina, L., Rossi, R.L., Querin, L., Wanke, V. & Vanoni, M. A cell sizer network involving Cln3 and Far1 controls entrance into S phase in the mitotic cycle of budding yeast. *J. Cell Biol.* **167**, 433–443 (2004).

5

a) Rabitsch KP, Toth A, Galova M, Schleiffer A, Schaffner G, et al. (2001) A screen for genes required for meiosis and spore formation based on whole-genome expression. Curr Biol 11: 1001–1009.
b) Jorgensen P, Nishikawa JL, Breitkreutz BJ, Tyers M (2002) Systematic identification of pathways that couple cell growth and division in yeast. Science 297: 395–400.
c) Costanzo M, Nishikawa JL, Tang X, Millman JS, Schub O, Breitkreuz K, Dewar D, Rupes I, Andrews B, Tyers M (2004) CDK activity antagonizes Whi5, an inhibitor of G1/S transcription in yeast. Cell 117: 899–913.
d) De Bruin RA, McDonald WH, Kalashnikova TI, Yates, J 3rd, Wittenberg C (2004) Cln3 activates G1-specific transcription via phosphorylation of the SBF bound repressor Whi5. Cell 117: 887–898.

6

a) Costanzo M, Nishikawa JL, Tang X, Millman JS, Schub O, Breitkreuz K, Dewar D, Rupes I, Andrews B, Tyers M (2004) CDK activity antagonizes Whi5, an inhibitor of G1/S transcription in yeast. Cell 117: 899–913.
b) De Bruin RA, McDonald WH, Kalashnikova TI, Yates, J 3rd, Wittenberg C (2004) Cln3 activates G1-specific transcription via phosphorylation of the SBF bound repressor Whi5. Cell 117: 887–898.

7

a) Nugroho TT, Mendenhall MD (1994) An inhibitor of yeast cyclin-dependent protein kinase plays an important role in ensuring the genomic integrity of daughter cells. Mol Cell Biol 14: 3320–3328.
b) Lengronne A, Schwob E (2002) The yeast CDK inhibitor Sic1 prevents genomic instability by promoting replication origin licensing in late G1. Mol Cell 9: 1067–1078.

8

a) Nugroho TT, Mendenhall MD (1994) An inhibitor of yeast cyclin-dependent protein kinase plays an important role in ensuring the genomic integrity of daughter cells. Mol Cell Biol 14: 3320–3328.
b) Tyers M (1996) The cyclin-dependent kinase inhibitor p40SIC1 imposes the requirement for Cln G1 cyclin function at Start. Proc Natl Acad Sci USA 93: 7772–7776.
c) Sanchez-Diaz A, Gonzalez I, Arellano M, Moreno S (1998) The Cdk inhibitors p25(rum1) and p40(SIC1) are functional homologues that play similar roles in the regulation of the cell cycle in fission and budding yeast. J Cell Sci 111: 843–851.
